# Molecular Phylogeny and Taxonomy of the Genus *Spumella* (Chrysophyceae) Based on Morphological and Molecular Evidence

**DOI:** 10.3389/fpls.2021.758067

**Published:** 2021-10-26

**Authors:** Minseok Jeong, Jong Im Kim, Seung Won Nam, Woongghi Shin

**Affiliations:** ^1^Department of Biology, Chungnam National University, Daejeon, South Korea; ^2^Nakdonggang National Institute of Biological Resources, Sangju-si, South Korea

**Keywords:** Chrysophyceae, heterotrophic, leucoplast, new species, phylogeny, *Spumella*, stomatocyst

## Abstract

The genus *Spumella*, established by Cienkowsky in 1870, is characterized by omnivory, two (rarely three) flagella, a short stick-like structure beneath the flagella, a threadlike stalk, cell division via constriction and cyst formation. Since the first phylogenetic study of *Spumella*-like flagellates, their paraphyly has consistently been shown, with separation into several genera. More recently, *Spumella* was carefully investigated using molecular and morphological data to propose seven new species. Classification of this genus and knowledge of its species diversity remain limited because *Spumella*-like flagellates are extremely difficult to identify based on limited morphological characters. To understand the phylogeny and taxonomy of *Spumella*, we analyzed molecular and morphological data from 47 strains, including 18 strains isolated from Korean ponds or swamps. Nuclear SSU, ITS and LSU rDNA data were used for maximum likelihood and Bayesian analyses. The molecular data divided the strains into 15 clades, including seven new lineages, each with unique molecular signatures for nuclear SSU rRNA from the E23-2 to E23-5 domains, the spacer between the E23-8 and E23-9 domains of the V4 region and domain 29 of the V5 region. Our results revealed increased species diversity in *Spumella*. In contrast to the molecular phylogeny results, the taxa showed very similar cell morphologies, suggesting morphological convergence into simple nanoflagellates to enable heterotrophy. Three new species produced stomatocysts in culture. Aspects of stomatocyst morphology, including collar structure, surface ornamentation, and cyst shape, were very useful in differentiating the three species. The general ultrastructure of *Spumella bureschii* strain Baekdongje012018B8 and *S. benthica* strain Hwarim032418A5 showed the typical chrysophyte form for the leucoplast, a vestigial chloroplast surrounded by four envelope membranes, supporting the hypothesis that *Spumella* evolved from a phototroph to a heterotroph via the loss of its photosynthetic ability. Seven new species are proposed: *S. benthica*, *S. communis*, *S. longicolla*, *S. oblata*, *S. rotundata*, *S. similis*, and *S. sinechrysos*.

## Introduction

The genus *Monas* was established by Müller (1773), who originally included three species: *Monas termo*, *M. lens*, and *M. mica*. At that time, members of the genus were delineated by being inconspicuous, mostly simple, translucent, and small in size. The first two species were transferred to *Oikomonas* and *Heteromita* (Kent, 1880) and are now known as *Oikomonas termo* and *Bodo lens*. Between creation of the genus and the middle of the nineteenth century, researchers described many *Monas* species (Müller, 1786; Ehrenberg, 1838; Dujardin, 1841; Pritchard, 1845, 1861; Perty, 1852; Fresenius, 1858; James-Clark, 1868). In 1838, Ehrenberg defined the genus using negative characteristics such as the lack of a tail, lip, stigma, and colony formation and added 26 new species. Dujardin (1841) redefined the genus more specifically as naked, round or oblong in shape, and possessing a flagellum and added 12 new species. James-Clark (1868) presented more details of two *Monas* species than provided in any previous description. Even then, the genus *Monas* was not well defined and was recognized as an assemblage of diverse protistan organisms.

In 1870, Cienkowsky established the new genus *Spumella* based on his detailed observations, characterizing the genus by its heterotrophy (feeding on algae, fungi, and starch grains), spherical or oval shape, two (rarely three) flagella, short stick-like structure beneath the flagella, one or two contractile vacuoles, short threadlike stalk, cell division via constriction at any point of the cell and cyst formation. Later, the type species, *Spumella vulgaris*, was considered the same as *Monas guttula* Ehrenberg (Stein, 1878; Kent, 1880) and finally synonymized as *M. guttula* (Reynold, 1934). Kent (1880) listed 26 *Monas* species and two *Spumella* species simultaneously and established a new *Monas*-like genus, *Physomonas*, described as a stalked monad possessing two unequal flagella. Three years later, Bütschli (1883) treated the genera *Spumella* and *Physomonas* as synonyms of *Monas*. In the early twentieth century, Pascher (1912) established another new genus, *Heterochromonas*, which is a counterpart of photosynthetic *Ochromonas*, and included two species previously known as *Monas* and *Spumella* species: *Heterochromonas vivipara* and *H. vulgaris*. Skuja (1939, 1948, 1956) followed Pascher’s classification system and added 10 new *Heterochromonas* species. Bourrelly (1957) restricted this genus to species that reliably produce endogenous cysts and designated *H. vivipara* as a lectotype. To date, more than 100 species of *Monas*-like flagellates (*Heterochromonas*, *Monas*, and *Spumella*) have been described (Supplementary Figure 1). According to Silva (1960), *Monas mica* is one of the originally described species and should be designated as the lectotype, but it has not observed since the time of first description. Therefore, there is no lectotype for the genus *Monas*. Silva (1960) suggested that the genera *Monas* and *Heterochromonas* should be abandoned due to the unknown identity of the lectotype and priority of the name *Spumella*, respectively. He included three species in the genus *Spumella*: *S. vulgaris* Cienkowsky, *S. vivipara* (Ehrenberg) Kent, and *S. beauchampii* (Hovasse) Silva. Most recently, studies revealed that *Spumella*-like flagellates are paraphyletic (Boenigk et al., 2005; Findenig et al., 2010; Grossmann et al., 2016). Therefore, considering this long taxonomic history of *Spumella*-like flagellates, it is thought that the genus *Spumella* should be defined in more detail.

*Spumella-*like flagellates are non-photosynthetic unicellular organisms with two unequal flagella that have a simple morphology, e.g., spherical or naked cells (Cienkowsky, 1870; Berglund et al., 2005; Boenigk et al., 2005; Lepère et al., 2006; Charvet et al., 2012). They are representative heterotrophic chrysophytes that underwent independent loss of photosynthetic ability in the chrysophyte lineage (Boenigk et al., 2005; Boenigk, 2008; Grossmann et al., 2016; Kim et al., 2020). As they are one of the major bacterivore groups in aquatic systems, their significance in carbon transfer from the microbial loop has been emphasized (Wylie and Currie, 1991; Sanders et al., 1994; Sherr and Sherr, 1994; Arndt et al., 2000; Boenigk and Arndt, 2002). Despite their important role in aquatic ecosystems and evolutionary change in nutritional mode from phototrophy to heterotrophy, relatively little attention has been given to their species diversity because of the high degree of morphological similarity. However, the genetic diversity of *Spumella*-like flagellates, including the genus *Spumella*, is high, suggesting that they are not a monophyletic lineage (Boenigk et al., 2005; Pfandl et al., 2009; Findenig et al., 2010). Recently, *Spumella*-like flagellates were separated into seven new genera based on nuclear SSU rDNA sequences, while the genus *Spumella* was finally epitypified (Findenig et al., 2010; Grossmann et al., 2016).

The genus *Spumella* is characterized by small cells (≤10 μm) with a spherical, sometimes elongated oval or oboval shape and a naked surface, two unequal flagella and colorlessness. Members of the genus lost their photosynthetic ability but still have a vestigial plastid (Preisig and Hibberd, 1983; Mylnikov et al., 2008; Grossmann et al., 2016) and retained their plastid genome (Beisser et al., 2017; Graupner et al., 2018; Dorrell et al., 2019; Kim et al., 2020). In particular, *Spumella vivipara* Kent, *S. sphaerophora* (Skuja) Mignot and *S. mior* (Skuja) Zhukov have strikingly large cell sizes of approximately 20 μm (Kent, 1880; Skuja, 1956; Mignot, 1977; Mylnikov and Mylnikova, 2005). Except for these three species, it is known that the vegetative cell morphologies of *Spumella* species are not useful in identification because of their small cell size and simple morphology. Instead, the morphology of the stomatocyst has been proposed as a useful characteristic feature for discriminating *Spumella* species (Findenig et al., 2010). However, only a few stomatocysts from *Spumella* species have been described because stomatocysts do not always develop under culture conditions (Yubuki et al., 2008; Findenig et al., 2010). The morphology of stomatocysts applies only to strains that develop stomatocysts; therefore, the identification of *Spumella* species based on morphological characters remains difficult.

Recently, to understand the genetic diversity of *Spumella* species, nuclear SSU, LSU, and 5.8S rDNA and the mitochondrial *cox*1 gene were used (Boenigk et al., 2005; Boenigk, 2008; Findenig et al., 2010; Grossmann et al., 2016; Bock et al., 2017). Based on molecular and morphological data, two species (*Spumella rivalis* Boenigk *et* Findenig and *S. lacusvadosi* Boenigk *et* Grossmann) were newly described, and *Monas bureschii* Valkanov was designated *Spumella bureschii* (Valkanov, 1925) Boenigk *et* Grossmann (Findenig et al., 2010; Grossmann et al., 2016). Compared with morphological characteristics, which are limited in their utility for species identification, molecular data are useful for identifying species on the basis of molecular signatures (Marin et al., 2003; Evans et al., 2007; Kynclova et al., 2010; Kim et al., 2013a, b; Skaloudova and Skaloud, 2013). The molecular signatures could be interpreted in the same way as generally accepted morphological criteria for classification (Marin et al., 2003), and they contain a similar phylogenetic signal and yield results largely congruent with phylogenetic relationships inferred using molecular data (Kynclova et al., 2010; Kim et al., 2013a, b; Skaloudova and Skaloud, 2013; Tragin et al., 2017).

The taxonomic definition of the genus *Spumella*, which has received much attention for a long time, has changed gradually over time. The currently recognized genus *Spumella* differs from that originally described by Cienkowsky (1870) in terms of various characters, and phylogenetic analysis of these *Spumella*-like flagellates using molecular data reveals that they are more diverse than previously expected. Despite the importance of *Spumella* as representative bacterivorous chrysophytes, their species diversity has been underestimated. To perform a taxonomic study of *Spumella*, we investigated 47 strains (including 18 strains isolated from Korea). Based on morphological and molecular data, we found high species diversity in *Spumella*, including seven new species. As key diagnostic characters for each species, we used stomatocyst morphology and molecular signatures, which were applied to each clade in the phylogenetic tree.

## Materials and Methods

### Cultures

Korean *Spumella* strains were collected from freshwater ponds or swamp sediments. We performed single-cell isolation using a Pasteur capillary pipette to establish unialgal cultures from field samples or subcultures. For unialgal clonal culture of *Spumella* strains, we performed two methods: single-cell isolation and dilution. For single-cell isolation, we picked up a single *Spumella* cell and a small aliquot of freshwater containing bacteria and then transferred them to each well of a 96-well plate. To make subcultures by the dilution method, 3 μl of field freshwater samples was mixed into 200 μl of AF-6 medium (Andersen et al., 2005) supplemented with 0.1, 0.2, or 0.5% Luria-Bertani (LB) medium (Miller, 1972). The LB broth was offered for bacterial growth. Bacteria from field freshwater samples were fed to *Spumella* cells as prey. When we observed the growth of *Spumella* cells, a single target cell was isolated and transferred to a new single-well plate by micropipette. Finally, all strains were grown in AF6 medium with a mixture of freshwater LB broth and uncharacterized bacteria from field freshwater samples. The strains were maintained at 17°C in a culture chamber in the dark. Information on the collection site and the GenBank accession number for each strain are listed in Supplementary Table 1.

### DNA Extraction, Amplification, Sequencing, and Alignment

The cells were harvested by centrifugation at 11,363 g for 5 min (11,000 rpm, model 5424, Eppendorf, Hamburg, Germany). Genomic DNA was extracted by a DOKDO-Prep^TM^ Blood Genomic DNA Purification Kit (ELPIS-Biotech Inc., Daejeon, Korea). Polymerase chain reaction (PCR) was performed for nuclear SSU, ITS and LSU rDNA using specific primers (Supplementary Table 2). PCR was performed in a total volume of 25 μl of the following: 1 μl of AccuPower^®^ PCR premix (Bioneer Co., Daejeon, Korea), 1 μl of forward primer, 1 μl of reverse primer, 2–10 μl of template DNA, and 12–20 μl of distilled water. The genes were amplified using a T100^TM^ Thermal Cycler (Bio-Rad Laboratories, Hercules, California, United States). The first denaturation was run at 94°C for 5 min, followed by 35 cycles of second denaturation at 94°C for 30 s, annealing at 42–52°C for 30 s-^–1^ min, extension at 72°C for 1–2 min, and a final extension at 72°C for 7 min, with a final hold at 12°C. All PCR products were purified using the Labopass^TM^ PCR Purification Kit or Labopass^TM^ Gel Purification Kit (Cosmogenetech Co., Seoul, Korea) following the protocol provided by the manufacturer. The purified PCR products were sequenced using an ABI PRISM^TM^ (3730xL, Perkin-Elmer Applied Biosystems, Foster City, California, United States). Sequence alignments were performed visually using the Genetic Data Environment (2.6) program (Smith et al., 1994).

### Molecular Data Analyses

The secondary structures of the nuclear (nr) SSU and LSU rRNA genes were aligned manually, with the alignment based on secondary structures of the nr SSU and LSU rRNA gene sequences of the dictyochophycean species *Apendinella radians* (Ali et al., 2001) as a guide in the Macgde (2.6) (Smith et al., 1994). As a molecular signature for *Spumella*-like flagellates, domain 8 from the V1 region of the nr SSU rRNA and D5 domain from the nr LSU rRNA gene were selected for identification of the genus *Spumella*. For discriminating species in the genus *Spumella*, the E23-2 to E23-5 domains, the spacer between the E23-8 and E23-9 domains of the V4 region and domain 29 of the V5 region of the nr SSU rRNA gene were selected.

### Phylogenetic Analyses

**FIGURE 1:**
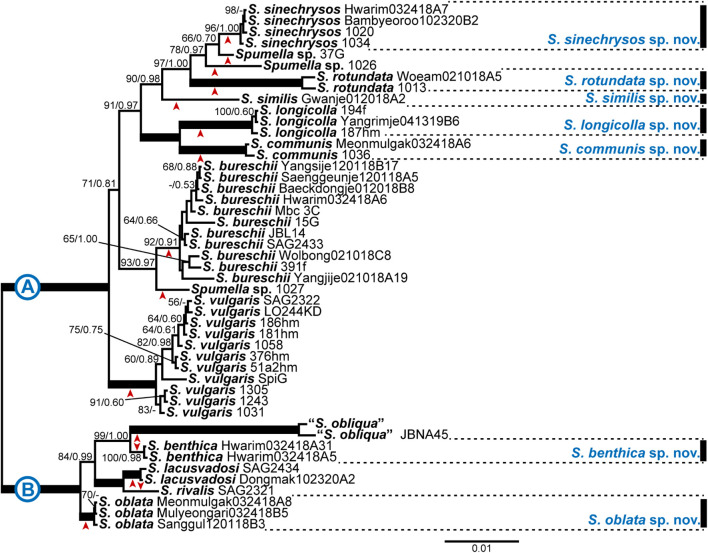
Bayesian tree of the genus *Spumella* based on combined nuclear SSU and LSU rDNA and ITS sequence data. The maximum-likelihood bootstrap values (MLBS values, left) and Bayesian posterior probabilities (PPs, right) are shown at each node. The arrow head below or above each node indicates each lineage; the scale bar indicates the number of substitutions/site; the thick line indicates full support (100% MLBS and 1.00 PP), and (–) denotes values < 50% for MLBS or 0.50 for PP.

The phylogenetic analysis was performed by using 1,625 nucleotides of the nr SSU rDNA from 158 chrysophycean taxa (Supplementary Figure 2). Three species (*Nanochloropsis granulata*, *Leukarachnion* sp., and *Synchroma grande*) were used as outgroup taxa (Grossmann et al., 2016; Pusztai and Škaloud, 2019). A combined dataset of 4,876 nucleotides (nr SSU = 1,667, nr LSU = 2,656, ITS1-5.8S-ITS2 region = 553) from 47 *Spumella* taxa was used for phylogenetic analysis (Figure 1 and Supplementary Table 1). For both datasets, conserved regions of genes were used only for phylogenetic analyses, and ambiguously aligned regions were excluded from phylogenetic analyses. Maximum likelihood (ML) was performed using RAxML version 8.2.10 (Stamatakis, 2014) with the general time-reversible plus gamma (GTR + I + G) model. We used 1,000 independent tree inferences, using the -# option of the program to identify the best tree. Maximum likelihood bootstrap (MLBS) values were calculated using 1,000 pseudoreplicates with the same substitution model. Bayesian analyses were performed using MrBayes version 3.7 (Ronquist et al., 2012), and the best-fitting model for the nucleotide dataset was selected by the Bayesian information criterion in jModelTest2 (Posada and Crandall, 1998), with the GTR + I + G model. Each analysis was performed using a Metropolis-coupled Markov chain Monte Carlo (MC3) approach, with 10,000,000 cycles for each chain. Trees were saved to a file every 1,000 cycles, and the burn-in point was identified graphically by tracking the likelihoods (Tracer version 1.7.1).^1^ The first 3,000 trees were discarded, and the remaining 7,001 trees were used to calculate the posterior probability (PP) of each clade. The trees were visualized using FigTree v.1.4.4.^2^

### Light Microscopy and Statistical Analysis

Culture strains were observed using an Axio Imager A2 microscope (Carl Zeiss Inc., Hallbergmoos, Germany) with differential interference contrast (DIC) optics. Images were captured with an AxioCam HRc (Carl Zeiss Inc.) and AxioCam 512 mono (Carl Zeiss Inc.) photomicrographic system connected to the microscope. Numerical values of morphological characters were obtained using photographic images of 25 cells (Table 1). The range of cell sizes was statistically analyzed using one-way ANOVA implemented in Excel 2016 to test for significantly different cell and flagellum sizes between *Spumella* taxa from Korea. A *p*-value under 0.05 was considered to indicate a statistically significant difference.

**TABLE 1:** Summary of vegetative cell and stomatocyst morphological characteristics of *Spumella*.

Taxon	Strain	Cell morphology						Stomatocyst morphology	
		Shape	Width (μm)	Length (μm)	Short flagellum (μm)	Long flagellum (μm)	Shape	Width (μm)	Length (μm)
*S. vulgaris* ^c^	199 hm	Spherical, elongated or posteriorly pointed shape	3.2–5.9 in diameter		0.7–1 times as long as the cell body	Twice as long as the cell body	Spherical with slightly conical collar and flat planar annulus around the pore	6.1–9.5 in diameter	
*S. benthica* ^a^	Hawrim032418A5	Circular(13), Widely elliptic(7), Widely obovate(1), Elliptic(3), Obovate(1)	2.7–4.8	3.3–5.4	1.4–3.0	2.8–6.7			
*S. bureschii* ^a^	Baekdongje012018B8	Circular(19), Widely elliptic(4), Elliptic(1), Obovate(1)	3.5–6.2	3.8–6.9	1.8–4.3	4.5–10.9			
*S. bureschii* ^b^	JBL14	Spherical, elongated or posteriorly pointed shape	2.9–7.4 in diameter		−	Up to 14.2	Spherical to slightly oval with rounded collar apex with outer collar margin connected to stomatocyst body	5.17–8.09 in diameter	
*S. communis* ^a^	Meonmulgak032418A6	Circular(17), Very widely obovate(1), Widely elliptic(5), Widely obovate(2), Elliptic(2)	2.4–4.9	3.0–5.4	1.6–4.2	4.2–11.0			
*S. lacusvadosi* ^a^	Dongmak102320A2	Circular(17), Very widely obovate(5), Very widely ovate(2), Widely elliptic(1)	2.2–4.7	2.9–4.8	1.0–3.2	3.1–7.7			
*S. lacusvadosi* ^b^	JBNZ39	Spherical, elongated or posteriorly pointed shape	1.5–7.1 in diameter		–	0.7–10.4			
*S. longicolla* ^a^	Yangrim041319B6	Circular(23), Widely elliptic(2)	3.7–8.5	4.0–9.1	1.6–3.1	4.1–9.8	Spherical (15) with cylindrical, long and thick collar	5.7–6.8	5.0–6.3
*S. oblata* ^a^	Sanggul120118B3	Circular(21), Widely elliptic(2), Elliptic(2)	2.5–4.3	2.5–4.9	1.5–3.4	2.4–7.9	Oblate (10) with thickened rim of round marginal collar apex	4.9–5.6	3.9–4.5
*S. rotundata* ^a^	Woeam021018A5	Circular(21), Widely elliptic(3), Elliptic(1)	2.0–5.8	2.3–5.8	2.0–3.6	4.7–9.1	Spherical (11) with thickened rim of round marginal collar apex.	5.0–5.8	4.5–5.4
*S. rivalis* ^c^	AR4A6	Spherical, elongated or posteriorly pointed shape	2.7–3.9 in diameter		0.7–1 times as long as the cell body	2 times as long as the cell body	Spherical with low rounded marginal rim collar	3.91–6.1 in diameter	
*S. similis* ^a^	Gwanje012018A2	Circular(21), Widely elliptic(3), Widely obovate(1)	3.8–5.5	4.0–6.5	1.9–3.4	4.8–8.8			
*S. sinechrysos* ^a^	Hwarim032418A7	Circular(19), Widely elliptic(5), Widely obovate(1)	3.0–5.8	3.2–5.7	2.0–3.5	4.0–7.2			

*The numbers in parentheses indicate the investigated number of cells (This study,^a^ Findenig et al., 2010;^b^ Grossmann et al., 2016^c^).*

### Transmission Electron Microscopy

To prepare the whole-mount transmission electron microscopy (TEM) samples, cells were fixed in 2.5% glutaraldehyde for 1 h at 4°C, and approximately 50 cells per strain were mounted on a formvar-coated copper grid. For ultrathin sectioning, the cells were collected by centrifugation for 5 min at 11,363 g (11,000 rpm, Eppendorf centrifuge 5424). After removing the supernatant, pelleted cells were fixed in 2.5% glutaraldehyde with 0.05 M cacodylate for 1 h at 4°C. After three 10 min washes with 0.05 M cacodylate buffer, the cells were fixed in 1% OsO_4_ for 1 h at 4°C and washed with 0.05 M cacodylate buffer. Dehydration was carried out in an ethanol series (50, 60, 70, 80, 90%) with 10 min for each step and three 10 min changes of absolute ethanol. The cells were serially transferred to three solutions of propylene oxide with ethanol (ethanol:propylene oxide = 2:1, 1:1, 1:2) for 15 min at each step and three times to absolute propylene oxide. The cells were serially transferred to three solutions of resin with propylene oxide (resin:propylene oxide = 1:2, 1:1, 2:1) for 2 h at each step and stored overnight in pure resin. The following day, the cells were transferred into new pure resin and polymerized at 70°C for 48 h. The polymerized blocks were thin-sectioned at a thickness of 70 nm. Serial sections were mounted on formvar-coated copper grids and stained with 3% (w/v) uranyl acetate and lead citrate. The grids were viewed and photographed using a JEM-1400 Plus transmission electron microscope (JEOL Ltd., Tokyo, Japan) at 120 kV.

### Scanning Electron Microscopy

To record stomatocyst morphology, 100–200 stomatocysts per strain were mounted on 0.45 μm nylon membrane filters using single-cell isolation. The filters were mounted onto aluminum stubs with double-sided tape. The stubs were coated with platinum and viewed with a MIRA3 field emission scanning electron microscope (TESCAN Ltd., Brno, Czech Republic) at 10 kV.

## Results

### Phylogenetic Analysis

The phylogenetic analyses of Chrysophyceae based on nr SSU rDNA sequence data included nine orders (Supplementary Figure 2). The six orders of Chrysophyceae were well resolved as monophyletic, and other three orders (Chromulinales, Hydrurales, and Paraphysomonadales) got a low statistical support. The phylogenetic relationships between nine orders were not resolved. The Ochromonadales formed a monophyletic clade with high support values (MLBS = 91, PP = 1.00) and included the genus *Spumella*. Within the order Ochromonadales, the phylogenetic relationships among the genera were not fully resolved. The genus *Spumella* formed a monophyletic clade with high support values (MLBS = 100, PP = 1.00), but the phylogenetic relationships among species were not resolved, and *S. bureschii* was paraphyletic in the phylogeny based only on nr SSU rDNA gene sequence data.

Based on the combined dataset of nr SSU, ITS and LSU rDNA gene sequences, phylogenetic analyses of the genus *Spumella* revealed that the 47 strains were divided into 15 subclades, including five consisting of a single taxon, and seven subclades included newly established species with high support values (Figure 1). The phylogenetic tree was divided into two major clades (A and B) with high support values (MLBS = 100, PP = 1.00). The first clade (A) included 10 subclades with five new species (*Spumella sinechrysos, S. rotundata, S. similis, S. longicolla*, and *S. communis*). The Hwarim032418A7, Bambyeoroo102320B2, 1020 and 1034 strains were grouped into the new species *S. sinechrysos* (MLBS = 96, PP = 1.00). Woeam021018A5 and 1030 were grouped together as the new species *S. rotundata* (MLBS = 100, PP = 1.00), and *S. similis* formed a single-taxon lineage sister to *S. sinechrysos*, *Spumella* sp. 37G, *Spumella* sp. 1026 and *S. rotundata* (MLBS = 90, PP = 0.98). The 194f, Yangrim041319B6 and 187hm strains were grouped together as the new species *S. longicolla* with high support values (MLBS = 100, PP = 1.00) and showed sister relationships with the new species *S. communis* (MLBS = 100, PP = 1.00). *S. bureschii* included 11 strains (MLBS = 92, PP = 0.91) and showed sister relationships with the *Spumella* sp. 1027 strain (MLBS = 93, PP = 0.97). The species *S. vulgaris* included 11 strains and formed a monophyletic clade with high support values (MLBS = 100, PP = 1.00). The second clade (B) consisted of five taxa, including two new species. The new species *S. benthica* consisted of two strains (MLBS = 100, PP = 0.98) and showed sister relationships with two “*S. obliqua*” strains with high support values (MLBS = 99, PP = 1.00). Mulyeongari032418B5, Sanggul120118B3 and Meomulgak032418A8 were grouped together into the new species *S. oblata* with high support values (MLBS = 100, PP = 1.00). The two formerly described species (*S. rivalis* and *S. lacusvadosi*) showed sister relationships with “*S. obliqua*” and *S. benthica* (MLBS = 84, PP = 0.99).

### Molecular Signatures

**FIGURE 2:**
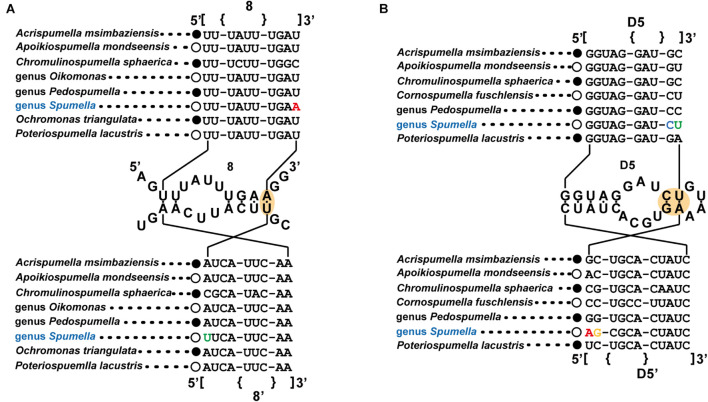
Molecular signatures of the nuclear SSU **(A)** and LSU **(B)** rRNA differentiating the genus *Spumella* from other non-photosynthetic chrysophytes. The secondary structure was constructed based on the genus *Spumella*. The nomenclature of nucleotides and base pairs depends on the polarity of the DNA: increasing numbers generally indicate the 5′–3′ direction. [] indicates the beginning and end of the stem, with {} indicating non-binding (loops, bulges) of the base pairs.

The molecular signatures of the nr SSU and LSU rRNA gene data were investigated with other representative non-photosynthetic lineages in Chrysophyceae to identify the molecular signatures unique to the genus *Spumella* (Figure 2). All *Spumella* species had unique sequences of “A:U” as the last base pair in domain 8 of the V1 region of nr SSU rRNA (Figure 2A) and “C:G” and “U:A” in domain D5 of the nr LSU rRNA gene (Figure 2B).

**FIGURE 3:**
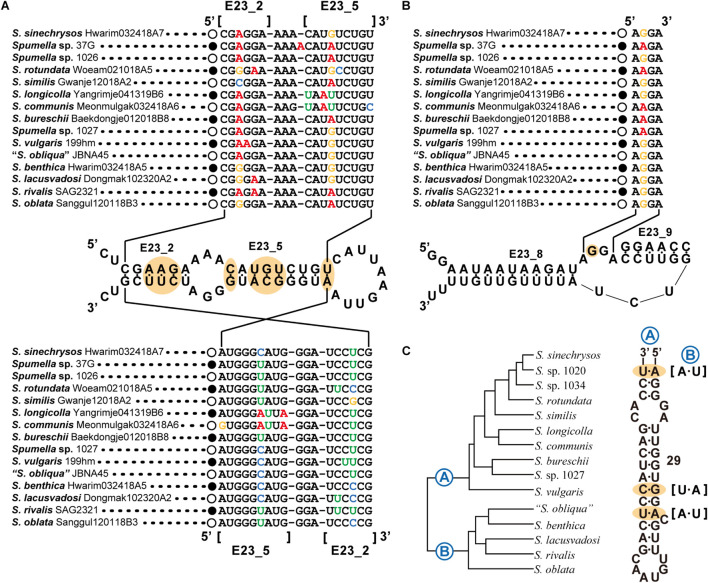
Molecular signatures of *Spumella* species using secondary structures from E23_2 to E23_5 **(A)**, E23_8 to E23_9 of the V4 region **(B)** and domain 29 of the V5 region **(C)** in the nuclear SSU rRNA gene sequence. The secondary structure was constructed based on *S. vulgaris*. The nomenclature of nucleotides depends on the polarity of the DNA: increasing numbers generally indicate the 5′–3′ direction. [] indicates the beginning and end of the stem region.

The nr SSU rRNA sequence combination including the E23-2 to E23-5 domain (Figure 3A), the spacer between E23-8 and E23-9 of the V4 region (Figure 3B) and domain 29 of the V5 region (Figure 3C) was selected as a molecular signature for the *Spumella* species. Each *Spumella* species had a specific molecular signature, and the signatures of new species were compared with those of closely related species. Base differences between *S. sinechrysos* and *Spumella* sp. 37G were found in E23-5 of the V4 region (G:C—A:U). Between *Spumella* sp. 1026 and *S. rotundata*, bases were different in E23-2 (A:U—G:C and G:C—A:U), E23-5 (A:U—G:C and U:G—C:G) and the spacer between E23-8 and E23-9 (G—A). *S. similis* was compared with *S. rotundata*, and bases were different in E23-2 (C:G—G:C and G:C—A:U), E23-5 (A:U—G:U and U:G—C:G) and the spacer between E23-8 and E23-9 (A—G). Two new species, *S. longicolla* and *S. communis*, were compared, and the bases were different in E23-5 (U:A—C:G). The base pair changes in the molecular signature region of *S. benthica* compared with that of “*S. obliqua*” JBNA45 were located at E23-2 (A:U—G:C). Base differences among three species, *S. lacusvadosi*, *S. rivalis* and *S. oblata*, were detected in E23-2 (G:C—A:U—G:C and A:U—A:U—G:C) and E23-5 (G:C—A:U—A:U). In the secondary structure of domain 29 of the V5 region, compensatory base pair changes (CBCs) occurred between the two major (A and B) clades of the phylogenetic tree of *Spumella* species (A:U—U:A, G:C—A:U, and A:U—U:A). In the secondary structure of the molecular signature region, several pairs of substitutions were CBCs or hemi-CBCs and conserved rRNA-base pairs of “G-C” but rarely changed to “G-U.” The base changes of each taxon are colored in Figures 2, 3.

### Morphological Characters

**FIGURE 4:**
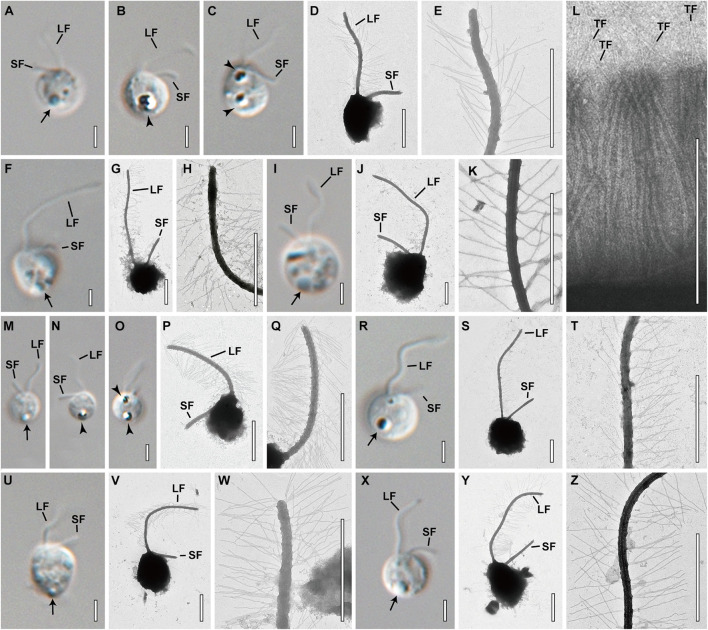
Light and transmission electron micrographs of *Spumella* species. Images showing each species having a long flagellum (LF) with two rows of mastigonemes, a terminal filament (TF) in the mastigonemes and a short flagellum (SF). Leucosin vesicle (arrow) and leucosin vesicles containing brilliant crystals (arrowhead) located on the anterior and posterior sides of the cell. **(A–E)** Image of *S. benthica* Hwarim032418A5. **(F–L)** Images of *S. communis* Meonmulgak032418A6. **(I–L)** Images of *S. longicolla* Yangrim041319B6. **(M–Q)** Images of *S. oblata* Sanggul120318B3. **(R–T)** Images of *S*. *rotundata* Woeam020118A5. **(U–W)** Images of *S*. *similis* Gwanje012018A2. **(X–Z)** Images of *S*. *sinechrysos* Hwarim032418A7. Scale bars = 2 μm.

The cell morphological characters of *Spumella* are summarized in Table 1, and the terminologies used for morphological description followed those of Radford (1986). Almost all *Spumella* species showed similar morphologies. The cell shapes of all *Spumella* species were circular to ovate, obovate or elliptic without an eyespot and cell surface ornamentation (Figure 4). The two flagella were unequal: the long flagellum had two rows of mastigonemes, but the short flagellum had no mastigonemes (Figures 4D,E,G,H,J,K,P,Q,S,T,V,W,Y,Z). The leucosin vesicle was positioned on the posterior side of the cell (Figures 4A,B,F,I,M,N,R,U,X) or on both the posterior and anterior sides of the cell when two leucosin vesicles were present in the cell (Figures 4C,O). In addition, one or two leucosin vesicles containing brilliant crystals were found only in *S. benthica* and *S. oblata* (Figures 4B,C,N,O).

### Stomatocyst

**FIGURE 5:**
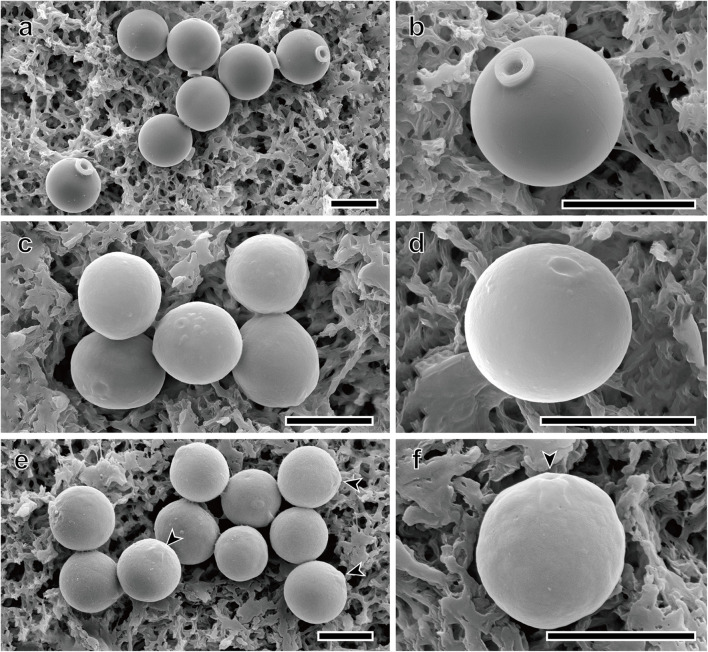
Scanning electron micrographs of three new *Spumella* species. **(a,b)** SEM images of the spherical stomatocyst of *S. longicolla* Yangrim041319B6. **(c,d)** SEM images of the oblate stomatocyst of *S. oblata* Sanggul120118B3. **(e,f)** SEM images of the spherical stomatocyst of *S. rotundata* Woeam020118A5. Arrowheads indicate a pore of the stomatocyst. Scale bars = 5 μm.

Only four species, *Spumella bureschii*, *S. longicolla*, *S. oblata*, and *S. rotundata*, encysted in culture, and the stomatocyst morphologies of these species were recorded using scanning electron microscopy (SEM) (Figures 5a–f). The terminology used for stomatocyst description followed that of Duff et al. (1995) and Wilkinson et al. (2001). The stomatocyst shape of *S. longicolla* was spherical without surface ornamentation (Figures 5a,b). The stomatocyst of *S. longicolla* had a distinct, long cylindrical collar, and sometimes the collar apex had several irregular grooves along its circumference (Figure 5b). The stomatocyst shape of *S. oblata* was oblate without surface ornamentation (Figures 5c,d). The collar of *S. oblata* was low and had a thickened rim with a rounded margin (Figure 5d). The stomatocyst shape of *S. rotundata* was spherical without surface ornamentation (Figures 5e,f). A distinct collar was not clearly observed, but there was a shallow raised and thickened region around the pore (Figure 5f). The outer margin of this region gently sloped down and was continuous with the stomatocyst body (Figure 5f).

### Ultrastructure

**FIGURE 6:**
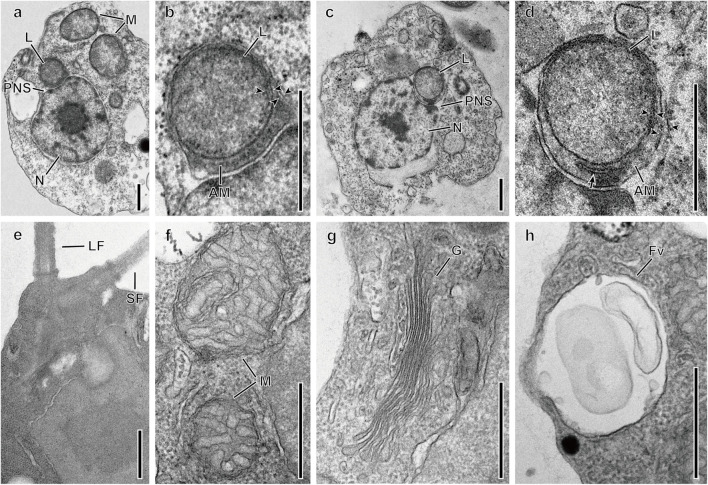
Transmission electron micrographs of *Spumella* species. **(a,b)** One leucoplast (L) of *S. bureschii* Baekdongje012018B8 located in the perinuclear space. **(c,d)** One leucoplast (L) of *S. benthica* Hwarim032418A5 located in the perinuclear space. **(e)** Flagellar apparatus of the long (LF) and short (SF) flagella. **(f)** Mitochondria with tubular cristae (M). **(g)** Golgi (G). **(h)** Food vacuole (Fv). Arrowheads indicate membrane envelops. AM, amorphous mass; PNS, perinuclear space; PR, periplastidial reticulum (arrow). Scale bars = 0.5 μm.

The general ultrastructure of two species, *Spumella bureschii* Baekdongje012018B8 and *S. benthica* Hwarim032418A5, as representatives of the two major clades in Figure 1, was observed with special attention to the vestigial plastid. One leucoplast was located in the perinuclear space between the inner and outer membranes of the nuclear envelope (Figures 6a–d) and was bound by four membranes. The outermost membrane was continuous with the outer membrane of the nucleus, while an amorphous mass including a membranous structure between the second and third membranes was visible. The two innermost membranes were closely appressed and formed an oval outline of the structure. The leucoplast did not show traces of a thylakoid (Figures 6a–d) and ranged from 0.43 to 0.48 μm in diameter. The two basal bodies were located perpendicular to each other, the long flagellum was directed forward, and the short flagellum was directed diagonally (Figure 6e). Swelling of the short flagellum was not found (Figure 6e). The typical structures of mitochondria, i.e., tubular cristae, a Golgi apparatus and a food vacuole, were observed (Figures 6f–h).

### Feeding

**FIGURE 7:**
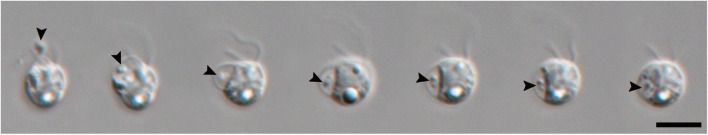
Serially captured LM images of four feeding phases of *S. rotundata*. Arrowheads indicate bacteria. Scale bar = 5 μm.

*Spumella* species consume bacteria through phagocytosis, and the four feeding phases of *S. rotundata* were recorded (Figure 7 and Supplementary Figure 3). The *Spumella* species adhered to the substratum during the feeding phases. The long flagellum caused water currents for feeding and detected the bacterium in the contact phase. After a food particle was detected, the long flagellum pressed the bacterium closely to the cell body in the processing phase. Then, the bacterium was ingested into the cell body using pseudopodia in the ingestion phase. Finally, in the refractory phase, the bulged part of the cell body involved in phagocytosis returned, and the hunted bacterium was positioned in the food vacuole of the cell.

### Description of New Species

#### *Spumella benthica* Jeong, M., Kim, J. I. et Shin, W. sp. nov.

DESCRIPTION: The species is colorless, non-scaled, single-celled and biflagellate without an eyespot (Figures 4A–E). Cells are circular, widely elliptic, widely obovate, elliptic or obovate, ranging in size from 2.7 to 4.8 × 3.3 to 5.4 μm. The two unequal emergent flagella are visible. The long flagellum has two rows of mastigonemes (Figures 4D,E) that are directed forward and waved or moved side to side, ranging in length from 2.8 to 6.7 μm. The short flagellum is directed diagonally or bent rearward and ranges in length from 1.4 to 3.0 μm. The leucosin vesicle is located in the posterior region of the cell (Figures 4A–C). One or two leucosin vesicles containing brilliant crystals are positioned in the cell (Figures 4B,C) and sometimes absent. The nr SSU rRNA molecular signature sequences are as follows:

E23-2 to E23-5 domain of the V4 region: CGGGGAAAACAUGUCUGU

E23-2′ to E23-5′ domain of the V4 region: AUGGGCAUGGGAUCCCCG

Spacer between the E23-8 and E23-9 domains of the V4 region: AGGA

HOLOTYPE: NNIBRPR19597, plastic TEM block Hwarim032418A5, deposited in the Nakdonggang National Institute of Biological Resources, Sangju, Korea (NNIBR).

TYPE LOCALITY: Freshwater, Hannam-ri, Namwon-eup, Seoguipo-si, Jeju-do, Korea (33°19′26.5^″^N, 126°39′31.9^″^E), 24 Mar. 2018.

ETYMOLOGY: The specific epithet “*benthica*” refers to the benthic habitat where this species is found.

#### *Spumella communis* Jeong, M., Kim, J. I. et Shin, W. sp. nov.

DESCRIPTION: The species is colorless, non-scaled, single-celled and biflagellate without an eyespot (Figures 4F–H). Cells are circular, very widely obovate, widely elliptic, widely obovate or elliptic, and ranging in size from 2.4 to 4.9 × 3.0 to 5.4 μm. The leucosin vesicle is located in the posterior region of the cell (Figure 4F). Two unequal emergent flagella are visible. The long flagellum has two rows of mastigonemes (Figures 4G,H) that are directed forward and waved or moved side to side, and ranging in length from 4.2 to 11.0 μm. The short flagellum is directed diagonally or bent rearward and ranges in length from 1.6 to 4.2 μm. The nr SSU rRNA molecular signature sequences are as follows:

E23-2 to E23-5 domain of the V4 region: CGAGGAAAGUAAUUCUGC

E23-2′ to E23-5′ domain of the V4 region: GUGGGAUUAGGAUCCUCG

Spacer between the E23-8 and E23-9 domains of the V4 region: AAGA

HOLOTYPE: NNIBRPR19601, plastic TEM block Meonmulgak032418A6, deposited in the Nakdonggang National Institute of Biological Resources, Sangju, Korea (NNIBR).

TYPE LOCALITY: Freshwater, Meonmulgak swamp, Seonheul-ri, Jocheon-eup, Jeju-si, Jeju-do, Korea (33°31′06.8^″^N 126°42′55.1^″^E), 24 Mar. 2018.

ETYMOLOGY: The Latin specific epithet “*communis*” refers to the ordinary shape of the cell.

#### *Spumella longicolla* Jeong, M., Kim, J. I. et Shin, W. sp. nov.

DESCRIPTION: The species is colorless, non-scaled, single-celled and biflagellate without an eyespot (Figures 4I–L). Cells are circular or widely elliptic, ranging in size from 3.7 to 8.5 × 4.0 to 9.1 μm. The leucosin vesicle is located in the posterior region of the cell (Figure 4I). Two unequal emergent flagella are visible. The long flagellum has two rows of mastigonemes (Figures 4J–L) that are directed forward and waved or moved side to side, ranging in length from 4.1 to 9.8 μm. The short flagellum is directed diagonally or bent rearward and ranges in length from 1.6 to 3.1 μm. The stomatocyst is spherical, ranges in size from 5.7 to 6.8 × 5.0 to 6.3 μm (*n* = 15), and has a smooth surface (Figures 5a,b). The collar of the stomatocyst is cylindrical and thick, ranging in height from 0.6 to 0.7 μm (*n* = 15) and in width from 1.0 to 1.8 μm (*n* = 14). The nr SSU rRNA molecular signature sequences are as follows:

E23-2 to E23-5 domain of the V4 region: CGAGGAAAAUAAUUCUGU

E23-2′ to E23-5′ domain of the V4 region: AUGGGAUUAGGAUCCUCG

Spacer between the E23-8 and E23-9 domains of the V4 region: AGGA

HOLOTYPE: NNIBRPR19599, plastic TEM block Yangrimje041319B6, deposited in the Nakdonggang National Institute of Biological Resources, Sangju, Korea (NNIBR).

TYPE LOCALITY: Freshwater, Yangrim pond, Jinyang-ri, Hampyeong-eup, Hampyeong-gun, Jeollabuk-do, Korea (35°05′16.6^″^N 126°29′57.6^″^E), 13 Apr. 2019.

ETYMOLOGY: The specific epithet “*longicolla*” derived from Latin “longi-” (= long) and “colla” (= collar) refers to the distinct, long collar of the stomatocyst.

#### *Spumella oblata* Jeong, M., Kim, J. I. et Shin, W. sp. nov.

DESCRIPTION: The species is colorless, non-scaled, single-celled and biflagellate without an eyespot (Figures 4M–Q). Cells are circular, widely elliptic or elliptic, ranging in size from 2.5 to 4.3 × 2.5 to 4.9 μm. The leucosin vesicle is located in the posterior region of the cell (Figures 4M–O). One or two leucosin vesicles containing brilliant crystals are positioned in the cell (Figures 4N,O) and sometimes absent (Figure 4M). Two unequal emergent flagella are visible. The long flagellum has two rows of mastigonemes (Figures 4P,Q) that are directed forward and waved or moved side to side, ranging in length from 2.4 to 7.9 μm. The short flagellum is directed diagonally or bent rearward, ranging in length from 1.5 to 3.4 μm. The stomatocyst is oblate, ranges in size from 4.9 to 5.6 × 3.9 to 4.5 μm (*n* = 10), and has a smooth surface (Figures 5c,d). The rim of the round marginal collar apex is slightly thickened (Figure 5d). The nr SSU rRNA molecular signature sequences are as follows:

E23-2 to E23-5 domain of the V4 region: CGGGGAAAACAUAUCUGU

E23-2′ to E23-5′ domain of the V4 region: AUGGGUAUGGGAUCCCCG

Spacer between the E23-8 and E23-9 domains of the V4 region: AGGA

HOLOTYPE: NNIBRPR19600, plastic TEM block Sanggul120118B3, deposited in the Nakdonggang National Institute of Biological Resources, Sangju, Korea (NNIBR).

TYPE LOCALITY: Freshwater, Sangdeung-ri, Booan-myeon, Gochang-gun, Jeollabuk-do, Korea (35°30′19.1^″^N 126°40′16.4^″^E), 01 December. 2018.

ETYMOLOGY: The Latin specific epithet “*oblate*” (= oblate) refers to the shape of the stomatocyst.

#### *Spumella rotundata* Jeong, M., Kim, J. I. et Shin, W. sp. nov.

DESCRIPTION: The species is colorless, non-scaled, single-celled and biflagellate without an eyespot (Figures 4R–T). Cells are circular, widely elliptic or elliptic, ranging in size from 2.0 to 5.8 × 2.3 to 5.8 μm. The leucosin vesicle is located in the posterior region of the cell (Figure 4F). Two unequal emergent flagella are visible. The long flagellum has two rows of mastigonemes (Figures 4S,T) that are directed forward and waved or moved side to side, ranging in length from 4.7 to 9.1 μm. The short flagellum is directed diagonally or bent rearward and ranges in length from 2.0 to 3.6 μm. The stomatocyst is spherical, ranges in size from 5.0 to 5.8 × 4.5 to 5.4 μm (n = 11), and has a smooth surface (Figures 5e,f). The rim of the round marginal collar apex is slightly thickened (Figure 5f). The collar margin gently slopes down and is continuous with the stomatocyst body (Figure 5f). The nr SSU rRNA molecular signature sequences are as follows:

E23-2 to E23-5 domain of the V4 region: CGGGAAAAACAUGCCUGU

E23-2′ to E23-5′ domain of the V4 region: AUGGGUAUGGGAUUCCCG

Spacer between the E23-8 and E23-9 domains of the V4 region: AAGA

HOLOTYPE: NNIBRPR19598, plastic TEM block Woeam021018A5, deposited in the Nakdonggang National Institute of Biological Resources, Sangju, Korea (NNIBR).

TYPE LOCALITY: Freshwater, Woeam pond, Moogo-ri, Moonnae-myeon, Haenam-gun, Jeollanam-do, Korea (34°37′45.1^″^N 126°18′16.4^″^E), 10 Feb. 2018

ETYMOLOGY: The Latin specific epithet “*rotundata* (= rounded)” refers to the stomatocyst shape.

#### *Spumella similis* Jeong, M., Kim, J. I. et Shin, W. sp. nov.

DESCRIPTION: The species is colorless, non-scaled, single-celled and biflagellate without an eyespot (Figures 4U–W). Cells are circular, widely elliptic or widely obovate, ranging in size from 3.8–5.5 × 4.0–6.5 μm. The leucosin vesicle is located in the posterior region of the cell (Figure 4U). Two unequal emergent flagella are visible. The long flagellum has mastigonemes (Figures 4V,W), which are directed forward and waved or moved side to side, ranging in length from 4.8 to 8.8 μm. The short flagellum is directed diagonally or bent rearward and ranges in length from 1.9 to 3.4 μm. The nr SSU rRNA molecular signature sequences are as follows:

E23-2 to E23-5 domain of the V4 region: CGCGGAAAACAUAUCUGU

E23-2′ to E23-5′ domain of the V4 region: AUGGGUAUGGGAUCCGCG

Spacer between the E23-8 and E23-9 domains of the V4 region: AGGA

HOLOTYPE: NNIBRPR19602, plastic TEM block Gwanje012018A2, deposited in the Nakdonggang National Institute of Biological Resources, Sangju, Korea (NNIBR).

TYPE LOCALITY: Freshwater, Gwanje pond, Bongeui-ri, Yongji-myeon, Kimje-si, Jeollabuk-do, Korea (35°52′18.0^″^N 126°57′15.2^″^E), 20 Jan. 2018

ETYMOLOGY: The Latin specific epithet *similis* (= similar) refers to the shape being extremely similar to that of other *Spumella* species

#### *Spumella sinechrysos* Jeong, M., Kim, J. I. et Shin, W. sp. nov.

DESCRIPTION: The species is colorless, non-scaled, single-celled and biflagellate without an eyespot (Figures 4X–Z). Cells are circular, widely elliptic or widely obovate, ranging in size from 3.0 to 5.8 × 3.2 to 5.7 μm. The leucosin vesicle is located in the posterior region of the cell (Figure 4X). Two unequal emergent flagella are visible. The long flagellum has two rows of mastigonemes (Figures 4Y,Z) that are directed forward and waved or moved side to side, ranging in length from 4.0 to 7.2 μm. The short flagellum is directed diagonally or bent rearward and ranges in length from 2.0 to 3.5 μm. The nr SSU rRNA molecular signature sequences are as follows:

E23-2 to E23-5 domain of the V4 region: CGAGGAAAACAUGUCUGU

E23-2′ to E23-5′ domain of the V4 region: AUGGGCAUGGGAUCCUCG

Spacer between the E23-8 and E23-9 domains of the V4 region: AGGA

Domain 29 of the V5 region: AGGGAUUGGUGGACGUU

Domain 29′ of the V5 region: GACUCCAUCAGCACCU

HOLOTYPE: NNIBRPR19603, plastic TEM block Hwarim032418A7, deposited in the Nakdonggang National Institute of Biological Resources, Sangju, Korea (NNIBR).

TYPE LOCALITY: Freshwater, Hannam-ri, Namwon-eup, Seoguipo-si, Jeju-do, Korea (33°19′26.5^″^N, 126°39′31.9^″^E), 24 Mar. 2018

ETYMOLOGY: The specific epithet “*sinechrysos*” was derived from Latin *sine*- (= without) and *chrysos* (= gold; the color of the chrysophycean plastid).

## Discussion

### Taxonomic Revision

In the early days after the first description of the genus *Monas* by Müller (1773), mostly heterotrophic and a few photosynthetic flagellates belonged to this genus (Müller, 1786; Ehrenberg, 1832, 1838; Perty, 1852). With advances in microscopy, observations of heterotrophic flagellates have become more detailed, and the limits of the genus have been changed slightly (Ehrenberg, 1838; Pritchard, 1861; Cienkowsky, 1870). Eventually, the genus *Monas* was divided into several *Monas*-related genera, and many species were transferred to other taxonomic ranks (Cienkowsky, 1870; Kent, 1880; Pascher, 1912; Grossmann et al., 2016). Furthermore, as Silva (1960) and Preisig et al. (1991) argued, the name *Monas* should be abandoned because the identity of its lectotype, *Monas mica*, designated by Diesing (1850), has not been confirmed since its original description by Müller (1773).

The genus *Spumella* was established by Cienkowsky (1870) and characterized by having a spherical or oval cell shape with two (or rarely three) unequal flagella, being colorless and heterotrophic (feeding on algae, fungi and starch grains), swimming or attaching to other substrata by a threadlike pedicle, and forming spherical cyst with short collar (Cienkowsky, 1870). He described *Spumella vulgaris* in detail and differentiated it from *Monas termo*, described by James-Clark (1868), according to the presence of three flagella, an anteriorly positioned nucleus, the absence of a protruding lip, and a difference in ingestion mode. When comparing the currently recognized *S. vulgaris* with the originally described *S. vulgaris*, there are also significant morphological differences. First, there is a difference in prey preference between the two species. While the current *S. vulgaris* is a bacterivore (Findenig et al., 2010), the original *S. vulgaris* appears to feed on a variety of prey, including eukaryotic cells (Cienkowsky, 1870). Second, there are large morphological differences between the two species. According to the original description of Cienkowsky (1870), *S. vulgaris* has two or rarely three flagella, a stick-like structure beneath the flagella, and a threadlike pedicle. However, all our strains showed two flagella and the absence of a stick-like structure and long threadlike pedicle. Lastly, cells of the originally described *S. vulgaris* divide into two or more parts by means of constriction (see Figures 52–54 on plate XXIV in Cienkowsky, 1870), but our strains divided into two by longitudinal cell division. A common feature of both species is the formation of cysts. Therefore, in light of these morphological differences, the currently recognized genus *Spumella* is clearly distinguished from the previously described genus.

### Cell Size

In this study, we performed morphological and molecular analyses to understand the phylogeny and taxonomy of *Spumella*. The Korean *Spumella* strains were colorless and naked on the cell surface and had two emergent flagella: one was a long flagellum with two rows of mastigonemes, and the other was a naked short flagellum. However, these morphological characters are typical of colorless chrysophycean lineages (Grossmann et al., 2016) and cause a problem in identification not only at the species level but also at the genus level. The morphological characters of *Spumella* species are also highly similar to each other and do not clearly differentiate the species. Even in the one-way ANOVA based on numerical values of vegetative cell morphology, the *p*-values were higher than 0.05 for all of the measured cell and flagellum size dimensions (Supplementary Figure 4), suggesting that these morphological characters are not significantly different among the species.

### Stomatocyst

The stomatocyst of *Spumella* species is a unique feature that is more reliable than vegetative cell morphology for distinguishing the species (Findenig et al., 2010). The three species among the seven new lineages also showed diagnostic characters related to stomatocyst morphology. The *Spumella* species with a collar include *S. bureschii* (rounded and thickened collar apex with the outer collar margin connected to the stomatocyst body), *S. rivalis* (collar with a rounded marginal rim), *S. sphaerophora* (thickened collar around the pore), and *S. hovassei* (approximately three tap-shaped collars) (Fiatte and Joyon, 1956; Skuja, 1956; Findenig et al., 2010). The slightly thickened, round marginal collar of the shallow raised area around the pore of *S. rotundata* is much more similar to those of *S. bureschii* and *S. spherophora* than to that of *S. rivalis*. However, the stomatocyst of *S. rotundata* is differentiated by its smaller diameter (5.0–5.8 μm) than those of *S. bureschii* (5.17–8.09 μm) and *S. sphaerophora* (9.0–11.0 μm). The cylindrical collar of *S. longicolla* is similar to that of *S. vulgaris* (Findenig et al., 2010) and *S. beauchampii* (Hovasse, 1943), but these two species have a flat planar annulus around the pore. In addition, the collar length of *S. longicolla* is approximately 2 times longer than that of *S. vulgaris* and is thicker than those of *S. vulgaris* and *S. beauchampii*. The low collar around the stomatocyst pore of *S. oblata* is similar to that of *S. rivalis*, but the shape in *S. oblata* is unique in being wider than the length. This is a striking characteristic feature that contrasts with the spherical shape of all previously reported *Spumella* stomatocysts, including those of *S. rivalis*. The newly described stomatocyst in this study also differed from the previously reported stomatocyst of *S. vivipara* (11.0 μm in diameter) in terms of size.

### Molecular Signature

Our phylogenetic tree based on the combined dataset showed the species diversity within the genus *Spumella* and divided the genus into 15 lineages with seven new species. The molecular signatures of the E23-2 to E23-5 domains, the spacer between E23-8 and E23-9 in the V4 region and domain 29 of the V5 region of nr SSU rRNA were found to differentiate the *Spumella* species. Due to the difficult identification of microalgal taxa with simple, ambiguous morphological characters, molecular features (DNA sequences or molecular signatures) have been efficiently used to describe different ranks, including at the species level (Marin et al., 2003; Kynclova et al., 2010; Choi et al., 2013; Kim et al., 2013a, b, 2017; Skaloudova and Skaloud, 2013). In particular, the V4 region of nr SSU rRNA is known as a useful genetic marker for understanding biological diversity in various protistan groups, including chrysophytes (Zimmermann et al., 2011; Bock et al., 2017; Tragin et al., 2017). The V4 region is also known as a hypervariable region (Ali et al., 2001; Tragin et al., 2017). The sequences in the molecular signature region are well conserved within species but variable among species in the genus *Spumella*. Furthermore, the molecular signatures are largely congruent with the phylogenetic relationships and helpful in delimiting *Spumella* species. Among *Spumella*-like flagellates, members of the genus *Spumella* had two unique molecular signatures in domain 8 of the V1 region of nr SSU rRNA and the D5 domain of nr LSU rRNA. Therefore, the genus *Spumella* can be easily distinguished from other *Spumella*-like flagellates by using these specific molecular signatures.

### Taxonomy of *Spumella* Species

Since the description of the genus *Spumella* by Cienkowsky (1870), many taxonomists have observed the morphological characteristics of *Spumella* under a light microscope or transmission electron microscope (Hovasse, 1943; Skuja, 1948; Silva, 1960; Mignot, 1977; Tanichev, 1993). Recent taxonomic studies have used both molecular and morphological data, showing that the species *Spumella vulgaris*,*S. bureschii*, *S. rivalis*, and *S. lacusvadosi* are well established as separate taxa (Findenig et al., 2010; Grossmann et al., 2016), but the identities of most traditionally recognized species remain unclear. Some previously described *Spumella* species, for example, *S. maior*, *S. vivipara*, *S. gregaria*, *S. sphaerophora*, and *S. dinobryonis*, are approximately 2–10 times larger in cell size than other *Spumella* species, including species newly described in this study. Mignot (1977) reported that *S. sphaerophora* has leucoplasts with a stigma (see Figures 4 and 8 in that article) and feeds on eukaryotic algal cells. However, none of the *Spumella* species investigated here provided any evidence of a stigma. In addition, the size and shape of the leucoplast in *S. sphaerophora* species differs from those in all *Spumella* species, which bear a single, small, oval-shaped leucoplast. Therefore, *S. sphaerophora* may not belong to *Spumella*. The species *Spumella dinobryonis*, *S. gregaria, S. guttula*, and *S. termo* have been characterized as having an anterior flagellum 2.0–3.5 times longer than their cell body length and attaching to substrates by a relatively long pedicel (Skuja, 1948; Tanichev, 1993). Another single-celled, unscaled, and stalked heterotrophic biflagellate belongs to the genus *Physomonas*, as established by Kent (1880). According to Kent’s classification system, the genus *Physomonas* is not affiliated with the Spumellidae or Monadidae but with the Dendromonadidae, suggesting that organisms bearing a long pedicel may belong to neither the genus *Spumella* nor other *Spumella*-like flagellates. The other previously described species in the genus *Spumella*, *S. beauchampii*, was originally described as a member of the genus *Oikomonas* (Hovasse, 1943) but later transferred to the genus *Spumella* (Silva, 1960) based on morphological characteristics, such as its distinct parabasal body at the bottom of the two flagella and, surprisingly, few *Chlorella*-like green algae in its food vacuoles. Given these characteristics, the species *S. beauchampii* is not considered to belong to the genus *Spumella*.

### Feeding

The genus *Spumella* is a well-known genus of bacterivores in Chrysophyceae and may be adapted to high concentrations of bacteria in diverse aquatic environments (Holen and Boraas, 1991; Boenigk and Arndt, 2000, 2002). The feeding behavior of Korean *Spumella* was the same as that previously described and recorded in two chrysophyte genera, *Ochromonas* and *Spumella* (Boenigk and Arndt, 2000). According to previous studies, *Spumella* species could feed on bacteria in both benthic and pelagic environments (Boenigk and Arndt, 2000, 2002). When they are in a benthic environment, *Spumella* adhere to the substratum using a short, pointed plasma membrane or the posterior cell surface. The cell creates filter currents toward the cell surface, aided by the flagella, and enfolds a food particle with a pseudopodium, after which the ingested food particles move toward the posterior end of the cell. In all our *Spumella* species, we observed the same feeding behavior and food vacuoles in the cytoplasm (Figure 7).

### Ultrastructure

The general ultrastructural features of *Spumella benthica* Hwarim032418A5 and *S. bureschii* Baekdongje012018B8, representative species of the two major clades (A and B) in the tree, were the leucoplast in the perinuclear space, mitochondria with tubular cristae, and perpendicularly arranged basal bodies but lack of photoreceptors (stigma) and swelling on the short flagellum. These morphological features resemble those of other members of the previously described *Spumella* (Belcher and Swale, 1976; Preisig and Hibberd, 1983; Tanichev and Karpov, 1993; Mylnikov et al., 2008). The leucoplast, known as a vestigial plastid, has been reported in the colorless chrysophycean genera *Spumella, Anthophysa*, and *Paraphysomonas* and dictyochophycean genera *Pteridomonas* and *Ciliophrys* (Belcher and Swale, 1972, 1976; Mignot, 1977; Preisig and Hibberd, 1983; Sekiguchi et al., 2002). The leucoplast in colorless chrysophycean genera is encircled by four membranes without any trace of the thylakoid membrane and is located in the perinuclear space. The outermost membrane is continuous with the outer nuclear membrane, and the periplastidial reticulum is located between the third membrane and the inner two membranes. The leucoplast observed in this study showed the same structure in terms of the number of membrane layers, presence of the periplastidial reticulum, and absence of thylakoid traces. However, the recently described genera *Poteriospumella* and *Cornospumella* do not show clear ultrastructural evidence, with the leucoplast not appearing to be encircled by the nuclear outer membrane (Grossmann et al., 2016). According to genomic and transcriptomic studies (Beisser et al., 2017; Graupner et al., 2018; Olefeld et al., 2018; Dorrell et al., 2019; Kim et al., 2020), the leucoplast of non-photosynthetic Chrysophyceae was found to have a reduced plastid genome. The plastid genome and transcriptome data showed that the non-photosynthetic lineages of chrysophytes lost most of their photosynthesis-related genes and evolved from phototrophic lineages.

Here, we studied the taxonomy of the genus *Spumella* based on morphological and molecular data and constructed a molecular phylogeny based on multigene data and increased taxon sampling. We offered detailed images of feeding behavior and ultrastructural images of the leucoplast and stomatocyst. Based on this evidence, we found high species diversity in the genus *Spumella*, including seven new species, and suggested key molecular signatures that can be used in taxonomic treatment.

## Data Availability Statement

The datasets presented in this study can be found in online repositories. The names of the repository/repositories and accession number(s) can be found in the article/Supplementary Material.

## Author Contributions

MJ, JIK, and WS conceived and designed the experiments, performed LM image recording, DNA extraction, PCR amplification, and phylogenetic analysis, interpreted the data, and wrote the manuscript. MJ, SWN, and WS performed the TEM- and SEM-based image recordings. All authors contributed to the article and approved the submitted version.

## Conflict of Interest

The authors declare that the research was conducted in the absence of any commercial or financial relationships that could be construed as a potential conflict of interest.

## Publisher’s Note

All claims expressed in this article are solely those of the authors and do not necessarily represent those of their affiliated organizations, or those of the publisher, the editors and the reviewers. Any product that may be evaluated in this article, or claim that may be made by its manufacturer, is not guaranteed or endorsed by the publisher.

## Footnotes

1
http://tree.bio.ed.ac.uk/software/tracer/

2
http://tree.bio.ed.ac.uk/software/figtree/
