# Supplementary figures and images for: Molecular Phylogeny and Taxonomy of the Genus Spumella (Chrysophyceae) Based on Morphological and Molecular Evidence
Source: Front Plant Sci. 2021 Oct 26;12:758067. doi: 10.3389/fpls.2021.758067 (PMC8577464; doi:10.3389/fpls.2021.758067)

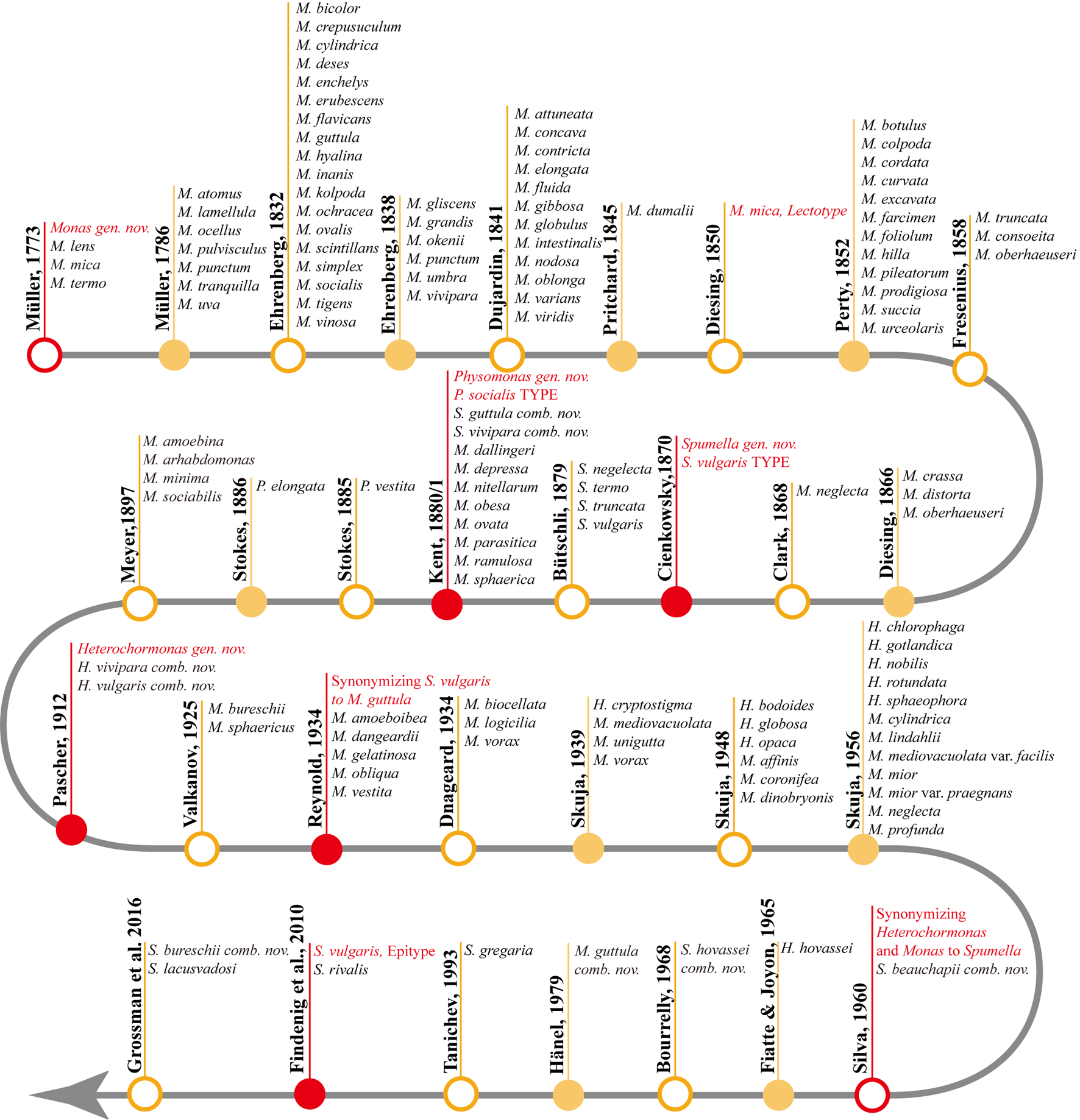

Supplement: Supplementary file 1 [file Image_1.TIF]

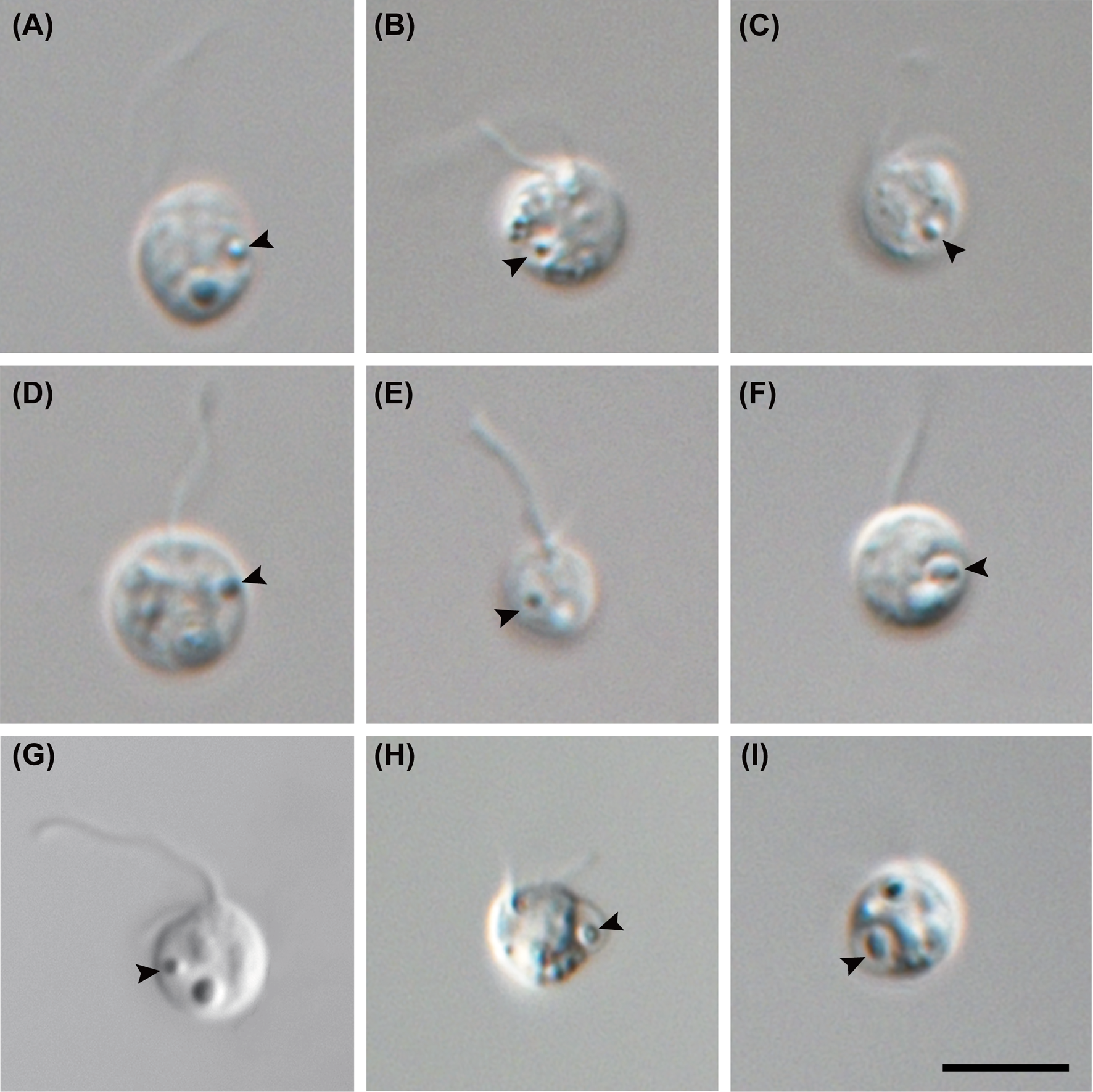

Supplement: Supplementary file 2 [file Image_2.TIF]

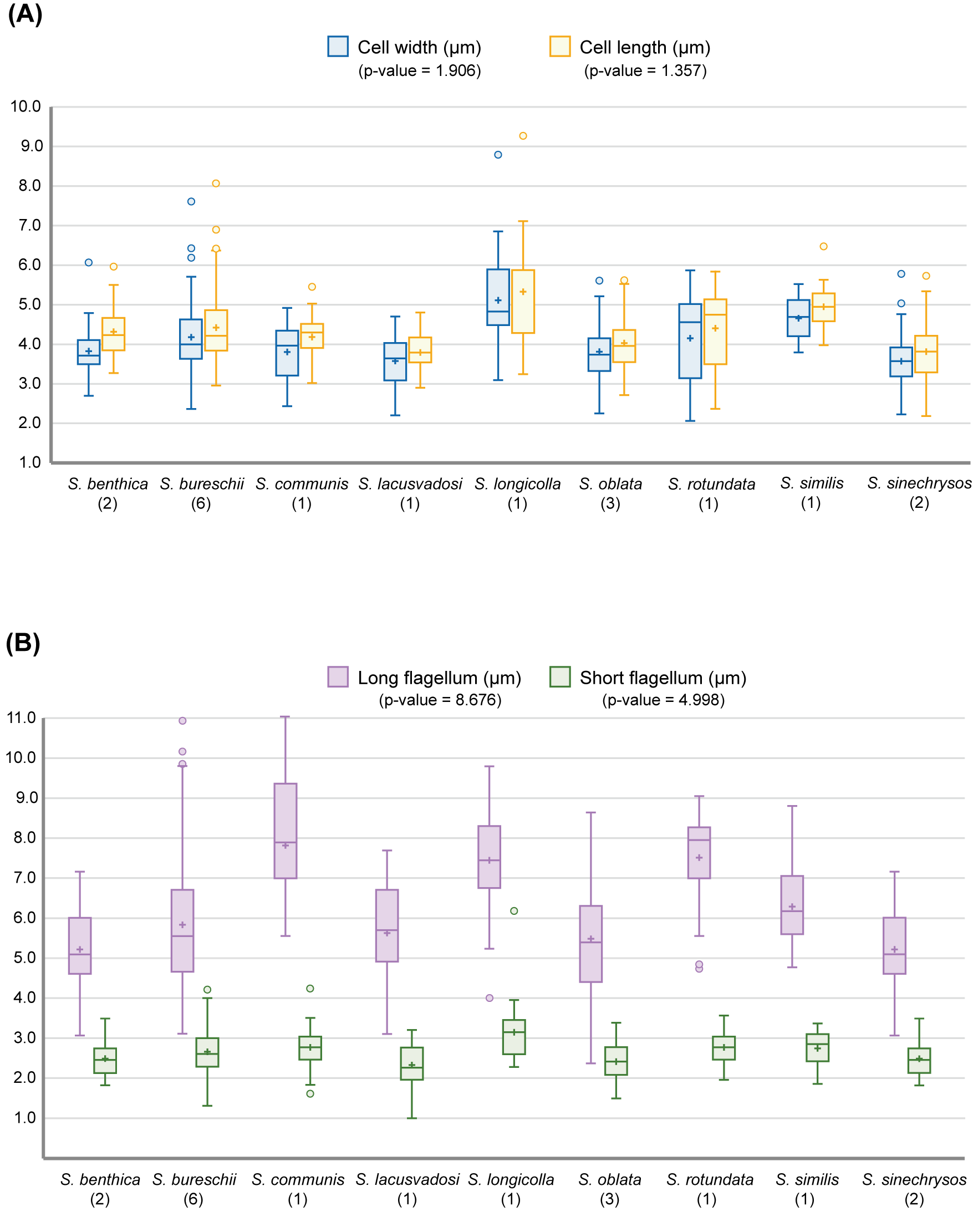

Supplement: Supplementary file 3 [file Image_3.TIF]

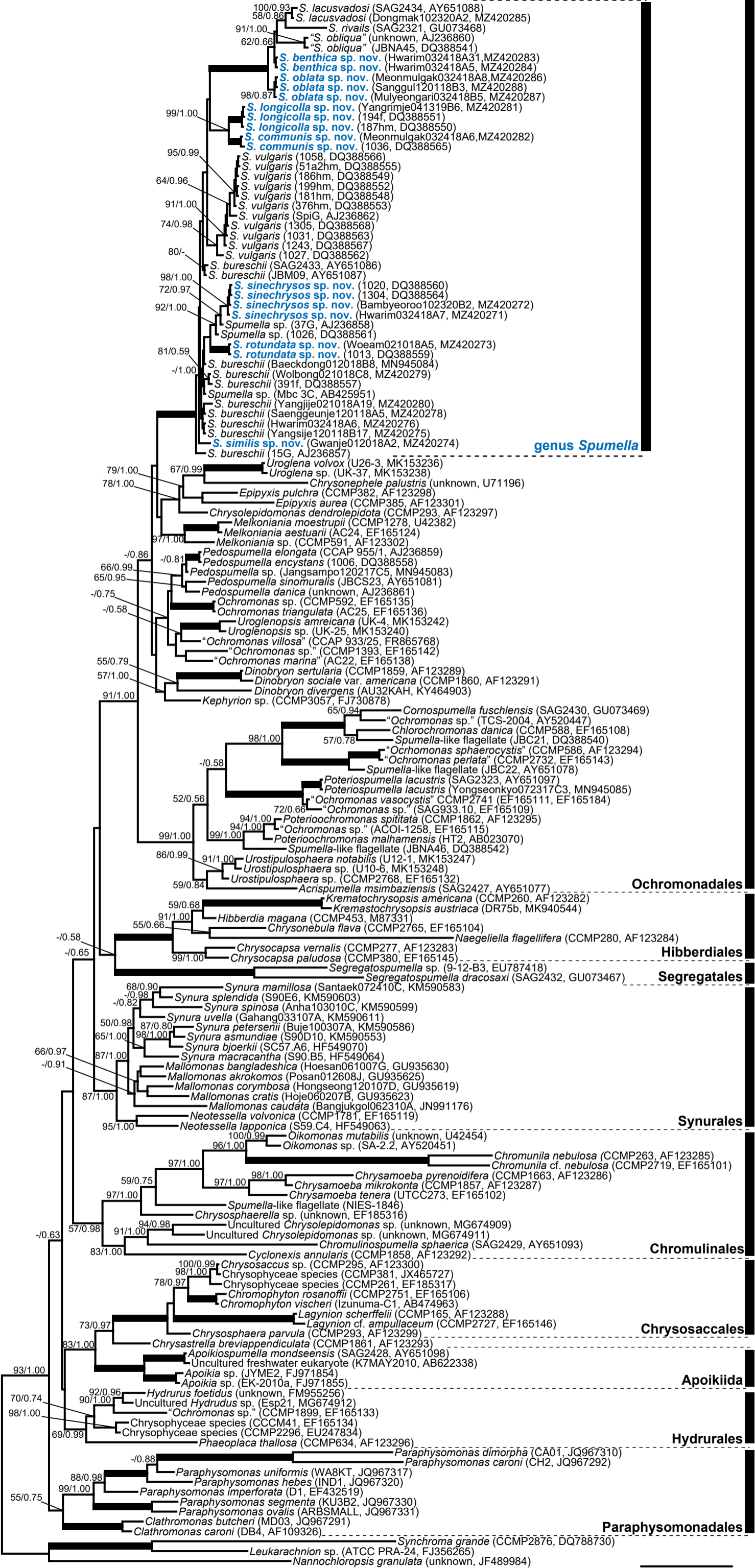

Supplement: Supplementary file 4 [file Data_Sheet_1.PDF]
