# Supplementary material for: Molecular Phylogeny and Taxonomy of the Genus Spumella (Chrysophyceae) Based on Morphological and Molecular Evidence
Source: Front Plant Sci. 2021 Oct 26;12:758067. doi: 10.3389/fpls.2021.758067 (PMC8577464; doi:10.3389/fpls.2021.758067)
Supplement: Supplementary file 5 [file Table_1.docx]

**Supplementary table 1.** Strains of genus *Spumella* used in this study and the GenBank accession numbers for their nuclear SSU, LSU rDNA and ITS gene sequences.

| Taxon | Strain | Collection site (GPS coordinates ) | Genbank accession number | | |
| --- | --- | --- | --- | --- | --- |
|  |  |  | SSU rDNA | ITS | LSU rDNA |
| ***Spumella*** |  |  |  |  |  |
| *S. vulgaris Cienkowsky* | LO244KD | Loibersbacher Teiche, Austria | - | - | KX100722 |
| *S. vulgaris Cienkowsky* | SpiG | unknown | AJ236862 | - | - |
| *S. vulgaris Cienkowsky* | 181hm | Davis Valley Pond, Davis Valley, Antarctica (82° 28'S, 50° 56'W) | DQ388548 | - | KX100700 |
| *S. vulgaris Cienkowsky* | 186hm | Coal Nunatak, Alexander Island, Antarctica (72° 07'S, 68° 32'W) | DQ388549 | KX150537 | KX100701 |
| *S. vulgaris Cienkowsky* | 199hm | Davis Valley Pond, Davis Valley, Antarctica (82° 28'S, 50° 56'W) | DQ388552 | EF577180 | KF697342 |
| *S. vulgaris Cienkowsky* | 376hm | Davis Valley Pond, Davis Valley, Antarctica (82° 28'S, 50° 56'W) | DQ388553 | EF577178 | KX100709 |
| *S. vulgaris Cienkowsky* | 51a2hm | Davis Valley Pond, Davis Valley, Antarctica (82° 28'S, 50° 56'W) | DQ388555 | - | KX100704 |
| *S. vulgaris Cienkowsky* | 1031 | Heywood Lake, Signy Island, Antartica (60° 42'S, 45° 36'W) | DQ388563 | EF577175 | KX100694 |
| *S. vulgaris Cienkowsky* | 1058 | Backslope, Signy Island, Antartica (60° 42'S, 45° 36'W) | DQ388566 | KX150536 | KX100697 |
| *S. vulgaris Cienkowsky* | 1243 | Heywood Lake, Signy Island, Antartica (60° 42'S, 45° 36'W) | DQ388567 | - | KX100698 |
| *S. vulgaris Cienkowsky* | 1305 | Heywood Lake, Signy Island, Antartica (60° 42'S, 45° 36'W) | DQ388568 | KX150535 | KX100699 |
| ***S. benthica* Jeong, Kim & Shin** | Hawrim032418A5 | Hannam-ri, Namwon-eup, Seoguipo-si, Jeju-do, Korea (33°19'26.5"N 126°39'31.9"E) | MZ420284 | MZ420302 | MZ420325 |
| ***S. benthica* Jeong, Kim & Shin** | Hwarim032418A31 | Hannam-ri, Namwon-eup, Seoguipo-si, Jeju-do, Korea (33°19'26.5"N 126°39'31.9"E) | MZ420283 | MZ420301 | MZ420324 |
| *S. bureschii* Boenigk & Grossmann | Baekdongje012018B8 | Baekdongje, Baekdong-ri, Limhoe-myeon, Jindo-gun, Jeollanam-do, Korea (34°22'39.3"N 126°11'00.4"E) | MN945084 | MZ420295 | MZ420318 |
| *S. bureschii* Boenigk & Grossmann | JBL14 | Puddle in Lunz, Austria (47°51'N, 15°03'E) | AY651086 | EF577172 | KF697329 |
| *S. bureschii* Boenigk & Grossmann | JBM09 | Puddle, Mondsee, Austria (47° 52'N, 13° 20'E) | AY651087 | KX150539 | KX100718 |
| *S. bureschii* Boenigk & Grossmann | Hwarim032418A6 | Hannam-ri, Namwon-eup, Seoguipo-si, Jeju-do, Korea (33°19'26.5"N 126°39'31.9"E) | MZ420276 | MZ420294 | MZ420317 |
| *S. bureschii* Boenigk & Grossmann | Saenggeunje120118A5 | Saenggeunje, Deoksan-ri, Seongnae-myeon, Gochang-gun, Jeollabuk-do, Korea (35°33'59.3"N 126°44'18.8"E) | MZ420278 | MZ420296 | MZ420319 |
| *S. bureschii* Boenigk & Grossmann | Wolbong021018C8 | Wolbongje, Laseong-ri, Haeri-myeon, Gochang-gun, Jeollabuk-do, Korea (35°28'04.3"N 126°28'51.8"E) | MZ420279 | MZ420297 | MZ420320 |
| *S. bureschii* Boenigk & Grossmann | Yangsije120118B17 | Yangsije, Gosoo-ri, Beaksoo-eup, Yeonggwang-gun, Jeollabuk-do, Korea (35°21'20.9"N 126°25'44.2"E) | MZ420275 | MZ420293 | MZ420316 |
| *S. bureschii* Boenigk & Grossmann | Yangjije021118A19 | Yangjije, Hwanea-ri, Masan-myeon, Haenam-gun, Jeollanam-do, Korea  (34°36'21.8"N 126°34'10.6"E) | MZ420280 | MZ420298 | MZ420321 |
| *S. bureschii* Boenigk & Grossmann | 391f | Forlidas Pond, Davis Valley, Antarctica (82° 27'S, 51° 21'W) | DQ388557 | EF577177 | - |
| ***S. communis* Jeong, Kim & Shin** | Meonmulgak032418A6 | Meonmulgak wetland, Seonheul-ri, Jocheon-eup, Jeju-si, Jeju-do, Korea (33°31'06.8"N 126°42'55.1"E) | MZ420282 | MZ420300 | MZ420323 |
| ***S. communis* Jeong, Kim & Shin** | 1036 | Heywood Lake, Signy Island, Antartica (60° 42'S, 45° 36'W) | DQ388565 | - | KX100696 |
| *S. lacusvadosi* Boenigk & Grossman | Dongmak102320A2 | Eodan-ri, Goojeong-myeon, Gangreung-si, Gangwon-do, Korea  (37°41'29.6"N 128°54'42.6"E) | MZ420285 | MZ420303 | MZ420326 |
| *S. lacusvadosi* Boenigk & Grossman | JBNZ39 | near Karangarua, New Zealand (43°37'S, 169°46'E) | AY651088 | KT697324 | KF697331 |
| ***S. longicolla* Jeong, Kim & Shin** | Yangrimje041319B6 | Yangrimje, Jinyang-ri, Hampyeong-eup, Hampyeong-gun, Jeollabuk-do, Korea (35°05'16.6"N 126°29'57.6"E) | MZ420281 | MZ420299 | MZ420322 |
| ***S. longicolla* Jeong, Kim & Shin** | 194f | Fossil Bluff, Alexander Island, Antartica (71° 00'S, 68° 00'W) | DQ388551 | EF577179 | KF697330 |
| ***S. longicolla* Jeong, Kim & Shin** | 187hm | Coal Nunatak, Alexander Island, Antarctica (72° 07'S, 68° 32'W) | DQ388550 | - | KX100702 |
| *“S. obliqua”* | - | unknown | AJ236860 | - | - |
| *“S. obliqua”* | JBNA45 | (Gold)fish pond, Valley of the Temple, Hawaii, Oahu (19° 33'N, 154° 53'W) | DQ388541 | EF577173 | KF697332 |
| ***S. oblata* Jeong, Kim & Shin** | Meonmulgak032418A8 | Meonmulgak wetland, Seonheul-ri, Jocheon-eup, Jeju-si, Jeju-do, Korea  (33°31'06.8"N 126°42'55.1"E) | MZ420286 | MZ420304 | MZ420327 |
| ***S. oblata* Jeong, Kim & Shin** | Mulyeongari032418B5 | Mulyeongari wetland, Sumang-ri, Namwon-eup, Seoguipo-si, Jeju-do, Korea (33°22'10.4"N 126°41'36.6"E) | MZ420287 | MZ420305 | MZ420328 |
| ***S. oblata* Jeong, Kim & Shin** | Sanggul120118B3 | Sangdeung-ri, Booan-myeon, Gochang-gun, Jeollabuk-do, Korea  (35°30'19.1"N 126°40'16.4"E) | MZ420288 | MZ420306 | MZ420329 |
| *S. rivalis* Boenigk & Findenig | AR4A6 | River Fuschler Ache (47°50'N, 13°16'E) | GU073468 | KF697328 | KF697344 |
| ***S. rotundata* Jeong, Kim & Shin** | Woeam021018A5 | Moogo-ri, Moonnae-myeon, Haenam-gun, Jeollanamdo, Korea (34°37'45.1"N 126°18'16.4"E) | MZ420273 | MZ420291 | MZ420314 |
| ***S. rotundata* Jeong, Kim & Shin** | 1013 | Changing Col, Signy Island, Antartica (60° 42'S, 45° 36'W) | DQ388559 | - | KX100690 |
| ***S. similis* Jeong, Kim & Shin** | Gwanje012018A2 | Bongeui-ri, Yongji-myeon, Kimje-si, Jeollabuk-do, Korea (35°52'18.0"N 126°57'15.2"E) | MZ420274 | MZ420292 | MZ420315 |
| ***S. sinechrysos* Jeong, Kim & Shin** | Bambyeroo102320B2 | Doogok-ri, Woocheon-myeon, Hoengseong-gun, Gangwon-do, Korea  (37°28'46.9"N 128°02'31.7"E) | MZ420272 | MZ420290 | MZ420313 |
| ***S. sinechrysos* Jeong, Kim & Shin** | Hwarim032418A7 | Hannam-ri, Namwon-eup, Seoguipo-si, Jeju-do, Korea (33°19'26.5"N 126°39'31.9"E) | MZ420271 | MZ420289 | MZ420312 |
| ***S. sinechrysos* Jeong, Kim & Shin** | 1020 | Backslope, Signy Island, Antarctica (60° 42'S, 45° 36'W) | DQ38850 | KX150541 | KX100691 |
| ***S. sinechrysos* Jeong, Kim & Shin** | 1034 | Mourraine Valley, Signy Island, Antartica (60° 42'S, 45° 36'W) | DQ388564 | - | KX100695 |
| *Spumella* sp. 1 | 1026 | Backslope, Signy Island, Antartica (60° 42'S, 45° 36'W) | DQ388561 | KX150542 | KX100692 |
| *Spumella* sp. 2 | 1027 | Changing Col, Signy Island, Antartica (60° 42'S, 45° 36'W) | DQ388562 | - | KX100693 |
| *Spumella* sp. 3 | 37G | unknown | AJ236858 | - | - |
