# Supplementary material for: Molecular Phylogeny and Taxonomy of the Genus Spumella (Chrysophyceae) Based on Morphological and Molecular Evidence
Source: Front Plant Sci. 2021 Oct 26;12:758067. doi: 10.3389/fpls.2021.758067 (PMC8577464; doi:10.3389/fpls.2021.758067)
Supplement: Supplementary file 6 [file Table_2.DOCX]

**Supplementary table 2.** Oligonucleotide primer sequences for the PCR amplification and sequencing of each gene.

| Gene | Pimer name | Sequences (5’ to 3’) | Direction | Reference |
| --- | --- | --- | --- | --- |
| Nuclear SSU | EukA | AAC CTG GTT GAT CCT GCC AGT | Forward | Medlin et al. (1988) |
|  | F-566 | CAG CAG CCG CGG TAA TTC C | Forward | Hadziavdic et al. (2014) |
|  | R-1200 | CCC GTG TTG AGT CAA ATT AAG C | Reverse | Hadziavdic et al. (2014) |
|  | EukB | TGA TCC TTC TGC AGG TTC ACC TAC | Reverse | Medlin et al. (1988) |
| Nuclear ITS | DF | CGC ACC TAC CGA TTG AAT | Forward | In this study |
|  | DR | CCT CCG CCT AGT TAT ATG CTT A | Reverse | In this study |
| Nuclear LSU | 28S_25F | ACC CGC TGA ATT TAA GCA TAT A | Forward | Jo et al. (2011) |
|  | 28S_1228F | CCT GAA AAT GGA TGG CGC | Forward | Jo et al. (2011) |
|  | 28S_1440R | TGCTGTTCACATGGAACCTTTC | Reverse | Jo et al. (2011) |
|  | 28S_2812R | GAT AGG AAG AGC CGA CAT CGA A | Reverse | Jo et al. (2011) |
