# Supplementary material for: Molecular Phylogeny and Taxonomy of the Genus Spumella (Chrysophyceae) Based on Morphological and Molecular Evidence
Source: Front Plant Sci. 2021 Oct 26;12:758067. doi: 10.3389/fpls.2021.758067 (PMC8577464; doi:10.3389/fpls.2021.758067)
Supplement: Supplementary file 7 [file Table_3.DOCX]

**Supplementary table 3.** Summary of the morphological characters and list of the *Spumella*-like flagellates.

| **Species name** | **Photo** | **Morphology** | | | | | |
| --- | --- | --- | --- | --- | --- | --- | --- |
|  |  | Cell  Width  (µm) | | Cell length  (µm) | Long flagellum  (µm) | Short flagellum  (µm) | Shape |
| **Genus *Acrispumella*** | | | | | | | |
| *Acrispumella msimbaziensis* Boenigk et Grossmann |   (Fig. 1F. strain JBAF33 = *Acrispumella msimbaziensis* n. gen. n. sp. in Grossmann et al. 2016) | 2-7.7 in diameter | | | Up to 17.4 |  | In Grossmann et al. (2016):  - Cells are typically elongated with posterior end.  - Two unequal flagella.  - Long flagellum have tripartite mastigonemes. |
| **Genus *Apoikiospumella*** | | | | | | | |
| *Apoikiospumella mondseeiensis* Boenigk et Grossmann |   (Fig. 1K. strain JBM08 = *Apoikiospumella mondseeiensis* n. gen. n. sp. in Grossmann et al. 2016) | 2-7.1 in diameter | | | Up to 12 |  | In Grossmann et al. (2016):  - Vegetative cells are mostly spherical, sometimes elongated or posteriorly pointed.  - Two unequal flagella.  - Long flagellum have tripartite mastigonemes.  - Second flagellum short and attached to the cell body. |
| **Genus *Cornospumella*** | | | | | | | |
| *Cornospumella fuschlensis* Boenigk et Grossmann |   (Fig. 4c. strain AR4D6 in Findenig et al. 2010)    (Fig. 5c. strain AR4D6 in Findenig et al. 2010)    (Fig. 1E. strain AR4D6 = *Cornospumella fuschlensis* n. gen. n. sp. in Grossmann et al. 2016) |  | | |  |  | In Findenig et al. (2010):  - Vegetative cells usually spherical, sometimes elongated or posteriorly pointed (2.8-4.7 µm in diameter).  - Two unequal flagella (Long: twice as long as cell body, Short: 0.7 to 1 times as long as the cell body).  - Stomatocyst have hook-like projections around the pore (4.35-5.7 µm in length, 3.96-5.45 µm in width).  - Some individual cysts have very low conical collar.  In Grossmann et al. (2016):  - Cells mostly spherical, sometimes elongated or posteriorly pointed (1.3-6.9 µm in diameter).  - Long flagellum (Up to 9.4 µm) have tripartite mastigonemes. |
| **Genus *Chromulinospumella*** | | | | | | | |
| *Chromulinospumella sphaerica* (Valkanov) Boenigk et Grosmann | ***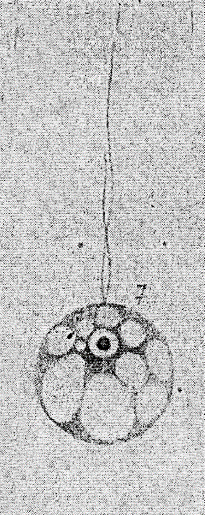***  (Fig. 7 *Monas sphaericus* n. sp. in Valkanov 1925)    (Fig. 1H. strain JBC27 = *Chromulinospumella sphaerica* n. gen. nov. comb. in Grossmann et al. 2016) |  |  | |  |  | In Valkanov (1925):  - Cells are spherical (4-6 µm in diameter).  - Two unequal flagella (Long: 2.5-3 times longer than cell length, Short: Almost equal to cell length).  In Grossmann et al. (2016):  - Cells are mostly spherical, sometimes elongated (2-8.6 µm in diameter).  - Long flagellum (Up to 15 µm) have tripartite mastigonemes. |
| **Genus *Heterochromonas*** | | | | | | | |
| *Heterochromonas bodoides* Skuja | 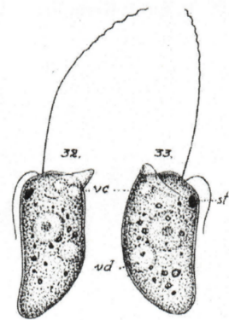  (Fig 32-33. *H. bodoides* comb. nov. st ;Stigma vc; contractile vacuoles, vd; digestive vacuoles. Plate XXXIV in Skuja 1948) | 10-12 | | 19-21 | 1.5 times longer than cell length | 0.33-0.5 times of the cell length | In Skuja(1948)  - Cell body shape is close to ovoid. The dorsal is arched and ventral is straight. The posterior of cell body is narrowed, curved and rounded.  - Having red stigma.  - Cell do not have large, protruding accumulation of leucosine. |
| *Heterochromonas chloropaga* Skuja | *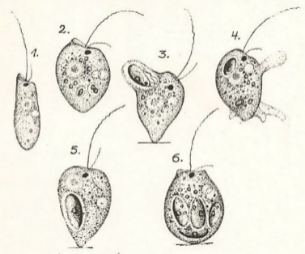*  (Fig. 1-6*. Heterochromonas chlorophaga* n. sp*.* 1 and 2; free-swimming, 3-6; fixed, 3, 5 and 6; some chlamydomonas taken in as food, 4; lobopodia. Plate LVI in Skuja 1956) | 5-12 | | 9-16 | Equal or slightly longer than cell body. | 0.14-0.20 times of the cell body. | In Skuja(1956):  - Cells are obovate or amoeboid.  - Can develop lobopodia.  - Having two unequal flagella.  - Having red stigma.  - Consume the bacteria, *Chlamydomonas* and colonial green algae as prey. |
| *Heterochromonas cryptostigma* Skuja | 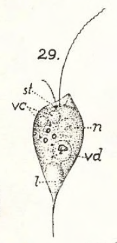  (Fig. 29. *Heterochromonas cryptostigma* Skuja. vc; pulsating vacuoles, vd; digestive vacuoles, st; stigma, I; leucosine, n; nucleus. Plate LV in Skuja 1956) | 5-8 | | 6-16 | 1.5-1.8 times longer than cell length. | 0.3 times of the cell length. | In Skuja(1939):  - Cells are obovate, ovate and rounded.  - Having two unequal flagella  - Having red stigma |
| *Heterochromonas globosa* Skuja | 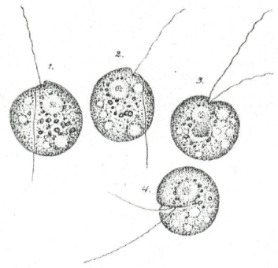  (Fig 1-4. *H. globosa* n. sp. 4 Top view Tab. XXXV in Skuja 1948) | 8-16 in diameter | | |  |  | In Skuja (1948):  - Cells are globose.  - Having longitudinal furrow  - Two flagella is almost equal.  - One flagellum is held forward while swimming and wriggled, the other is dragged along.  - Carotenoid bodies are sometimes present in the cell. |
| *Heterochromonas gotlandica* Skuja |  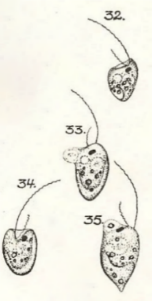  (Fig. 30-35. *H. gotlandica* n. sp. Different formation of the cells, 33 and 35 with a lobopodium and in this located digestive vacuole. Plate LV in Skuja 1956) | 4-7 | | 7-12 | Equal or slightly longer than cell length. | 0.20-0.25 times of the cell length. | In Skuja (1956):  - Cells are obovate with obliquely truncated anterior. Posterior often narrowed sharply rounded.  - Two unequal flagella.  - Having red stigma |
| *Heterochromonas nobilis* Skuja | *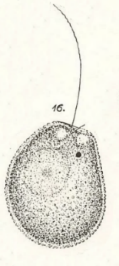*  (Fig. 16*. H. nobilis* n. sp. Plate LVI in Skuja 1956) | 25-28 | | 30-35 | Longer than cell body. | 0.1-0.2 times of the cell length. | In Skuja (1956):  - Cells are rounded-oval.  - Two unequal flagella.  - Having red stigma  - Sometimes a few carotenoid bodies scattered in the cell.  - Having clear line of the mouth. |
| *Heterochromonas opaca* Skuja | *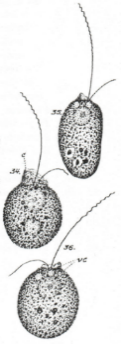*  (Fig 34-36. *H. opaca* n. sp. 35: young specimen just created by division, c ± formed plasma collar, vc contractile vacuoles. Tab. XXXIV in Skuja 1948) | 11-23 | | 16-23 | Equal or slightly longer than cell length. | 0.33-0.5 times of the cell length. | In Skuja (1948)  - Cells are globose. After division, cells are ellipsoidal, anterior is truncated with depression.  - Two unequal flagella. |
| *Heterochromonas polystica* Skuja | *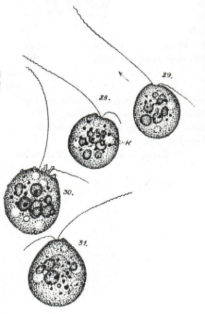*  (Fig 28-31. *H. polystica* n. sp. k reddish carotenoid bodies; the arrow in Fig. 29 shows the direction of movement with the stated position of the floating flagella. Tab. XXXIV in Skuja 1948) | 10-14 | | 12-17 | 1.5-2 times longer than cell body. | 0.14-0.2 of the cell length. | In Skuja (1948):  - Cells are globse. Slight protruding of anterior.  - Two unequal flagella.  - Having carotenoid bodies in the cell with various size.  - Consume various small algae as a prey.  - Do not have accumulation of luecosine. |
| *Heterochromonas rotundata* Skuja | *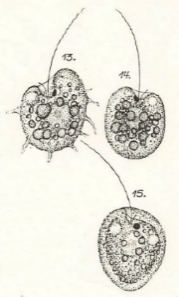*  (Fig. 13-15*. H. rotundata* n. sp. 13; rhizopodia. Plate LVI in Skuja 1956) | 15-18 | | 15-20 | Equal or 1.3 times longer than cell length | 0.1 times of the cell length. | In Skuja (1956):  -Cell are rounded-obovate, nearly spherical.  -Cell surface is warty.  - Two unequal flagella.  - Stretch out the short, irregular pseudopodia all over the body.  - Having red stigma |
| **Genus *Monas*** | | | | | | | |
| *Monas affinis* Skuja | 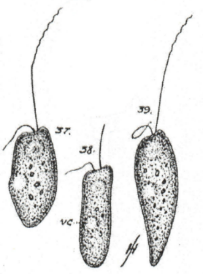  (Fig. 37-39 *M. affinis* n. sp. vc contractile vacuole.) | 5-8 | | 17-22 | Slightly longer than cell length. | 0.2-0.3 times of the cell length. | In Skuja (1948):  - Cells are cylindrical, ellipsoidal.  - Two unequal flagella.  - Lack noticeable accumulation of leucosine. |
| *Monas attenuata* Dujardin | 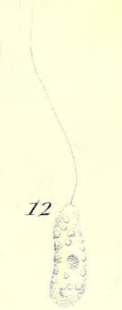  (Plate 3, Fig. 12 in Dujardin 1841) |  | | 16 |  |  | In Dujardin (1841):  - Cells are ovoid, narrowed both end, nodular.  - One flagellum  - It would be *Cercomonas*  - Marsh water |
| *Monas atomus* Müller | 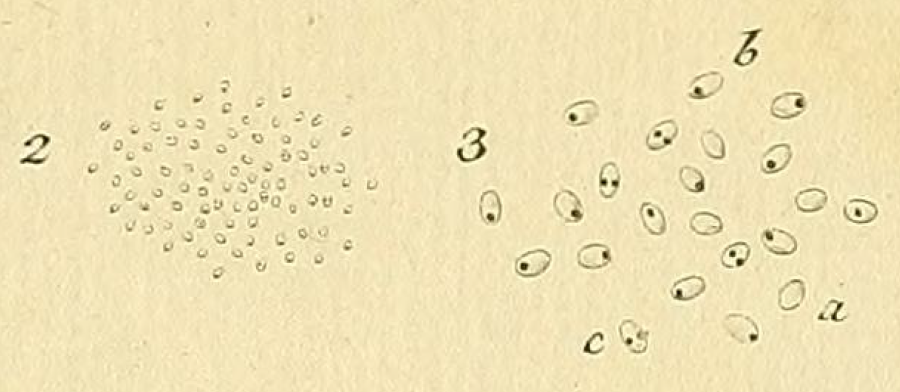  (Fig. 2. *M. atomus*, enlarged. Fig. 3. More enlarged, a. cell without dot, b. cell with one dot, c. cell with two dots. Plate 1 in Müller 1786) |  | |  |  |  | In Müller (1786):  - Cells |
| *Monas biocellata* Dangeard | 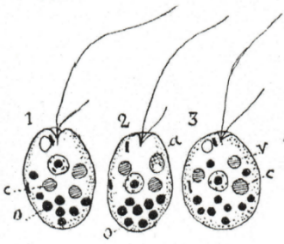  (Fig 1-3. *M. biocellata* n. sp. – with ingested algae a, chromidia c and liposome. Plate XXVIII in Dangeard 1934) |  | | 20 | 1-2 times longer than cell length |  | In Dangeard (1934):  - Cells are elliptic.  - Two unequal flagella.  - Having stigma.  - Consume the algae as a prey. |
| *Monas biocolor* Ehrenberg | 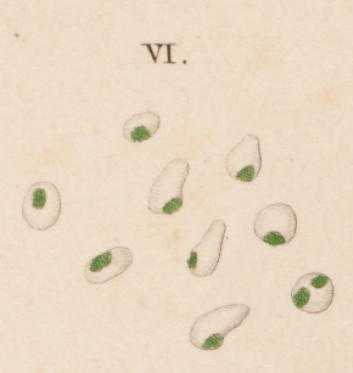  (Fig. 6. *M. biocolor.* 9 individuals of this monad are shown enlarged 290 times in different positions and states, the majority of which have only 1 green core. Plate 1 in Ehrenberg 1838) | 16.6 | |  |  |  | In Ehrenberg (1838):  - Cells are globular or oval.  - One or two green granules in the cell.  - Cells have wavering movement. |
| *Manas concava* Dujardin | The imgaes did not published. | 12.3 | |  |  |  | In Dujardin (1841):  - Cells are cicular body, concave on one side, convex on the other.  -Marsh water |
| *Monas constrica* Dujardin | The imgaes did not published. |  | | 20 |  |  | In Dujardin (1841):  - Cells are elongated with rounded back and bodies are thick. |
| *Monas coronifera* Skuja | 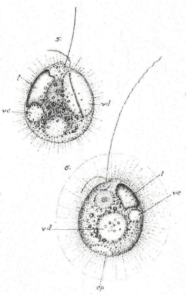  (Fig. 5-6 *M. coronifera* n. sp. 5 with incomplete, 6 with fully developed radial structured gelatinous envelope; l Leucosine accumulation, cp cytoproct, vc contractile vacuole, and vd digestive vacuole in FIG. 5 with an ingested bacterium.)  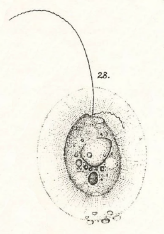  (Fig. 28. *M. coronifera* Skuja fa. The digestive residues expelled by the cytoproct have partially stuck to the back of the gelatinous envelope around the opening located here. Plate LVI in Skuja 1956) | 11-19 | | 13-23 | 1.5-2 times longer than cell length. | 0.2-0.33 times of the cell length. | In Skuja (1948):  - Cells are rounded, ovate with thick, radially structured gelatinous envelope  - Consume the bacteria as a prey, rarely unicellular algae.  - Cells are anchored using long pedicel.  - Having line of mouth.    In Skuja (1956):  - Cells are surrounded by gelatinous shell that is excreted from submicroscopic pores in peripheral of the cell. |
| *Monas crepusculum* Ehrenberg | 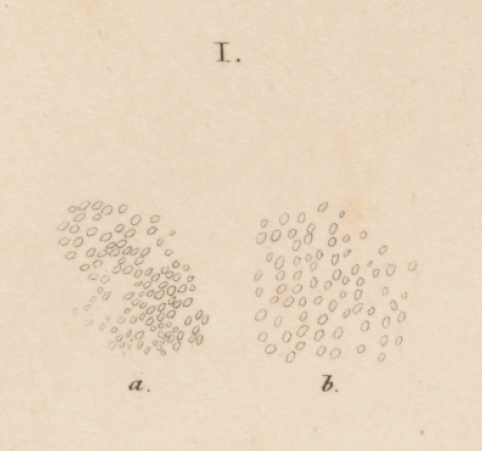  (Fig. 1. *M. crepusculum.* a. is enlarged 450 times in diameter, with the eye 9 inches from the object. b. is enlarged 820 times. Greater magnifications result in a loss of clarity and no gain in insight into the structure. With a 3000-fold enlargement in diameter, one sees them in the form like Fig. 2. b., But essentially not differently, only less clearly. Plate 1 in Ehrenberg 1838) |  | |  |  |  | In Ehrenberg (1838):  - Cells are spherical and colorless.  - Consume animal or fungal substance as a prey (carnivore). |
| *Manas cylindrica* Skuja | 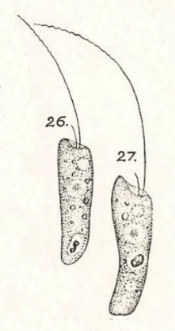  (Fig. 26-27. *M. cylindrica* n. sp. Plate LVI in Skuja 1956) | 3-6 | | 10-15 | 1.5-2 times longer than cell length | 0.06-0.1 times of the cell length. | In Skuja (1956):  - Cells are cylindrical to cylindric clavate.  - Two unequal flagella.  - Form slight lobopodia. |
| *Monas cylindrica* Ehrenberg | 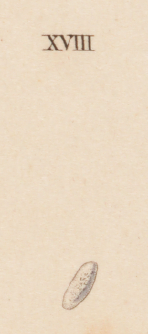  (Fig. 18. *M. cylindrica.* It represents the single shape drawn by Orenburg, which is 1/96 line long and enlarged 245 times. Plate 1 in Ehrenberg 1838) |  | | 20.8 |  |  | In Ehrenberg (1838):  - Cells are cylindrical and middle part of the cell is slightly swelling.  - Cells have swirling movement. |
| *Monas deses* Ehrenberg | 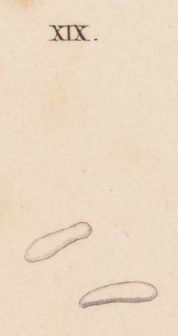  (Fig. 19. *Monas deses.* It is a specimen of the *Monas deses* in 2 positions. Size 1/100 line. Magnification 245 times in diameter. The drawing was made by me in Syrjanofskoi. Plate 1 in Ehrenberg 1838) | 0.25-0.3 of the cell length. | | 20 |  |  | In Ehrenberg (1838):  - Cells are conical, oblong and have a green color. |
| *Monas elongata* Dujardin | 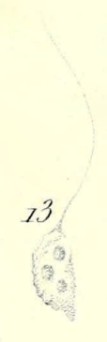  (Plate 3, Fig. 13 in Dujardin 1841) |  | | 20 | 40 |  | In Dujardin (1841):  - Cells are elongated, nodular and flexible.  - One flagellum  - Marsh water  - It would be a *Peranema*. |
| *Monas enchelys* | 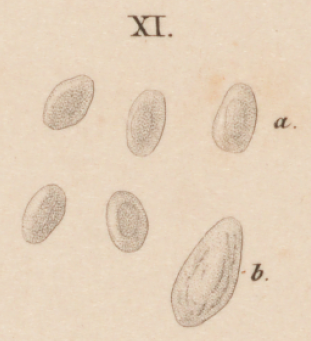  (Fig. 11. *M. enchelys.* a. are 5 individuals observed near Berlin, 1/80 line size, 290 times enlarged, b. is a single, 1/100 line size, enlarged 525 times from the Urals. The drawing should be somewhat larger, but it is measured, and the difference is a consequence of the different sensitivity of the eye to the sizes. I deliberately did not change it, but indicated the measurements. Plate 1 in Ehrenberg 1838) |  | | 20-25 |  |  | In Ehrenberg (1838):  - Cells are ovate, rounded.  - Cells move slowly and continuously. |
| *Monas erubescens* Ehrenberg | 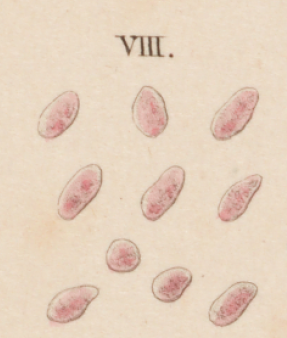  (Fig. 8. *M. erubescens.* There are 10 individuals of the *M. erubescens* in different sizes and positions enlarged 300 times. Further structure and developmental relationships are not observed. Plate 1 in Ehrenberg 1838) | 13.9 | |  |  |  | In Ehrenberg (1838):  - Cells are oval and have rose color.  - Cells move continuously and slowly. |
| *Monas flavicans* Ehrenberg | 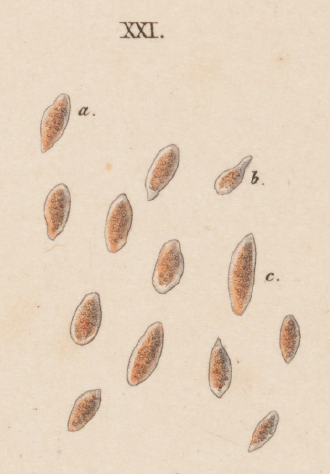  (Fig. 21. *Monas flavicans.* Among the group of 13 individuals shown, the pear-shaped or conical ones are the normal forms. The pointed end is the hind part of all. They swim in different directions and are of different sizes. The largest are 1/144 line in size and all enlarged 380 times. The variability of the form, reminiscent of *Astasia*, is not clearly arbitrary. Fig. a. is an individual who temporarily protrudes the part of the mouth a little. Fig. b. is an animal with its very thinned abdomen directed upwards. Fig. c. is an almost spindle-like elongated shape that occurs when the water droplets evaporate, not a permanent normal shape. Plate 1 in Ehrenberg 1838) | 0.5 of the cell length | | 13.8 |  |  | In Ehrenberg (1838):  - Cells are conical, inverted cone-shaped with sharp posterior body.  - Cells are yellowish and have continuous sliding movement. |
| *Monas fluida* Dujardin | 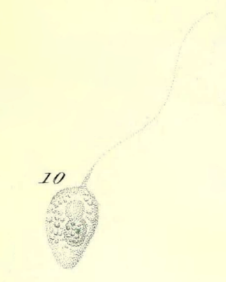  (Plate 4, Fig. 10. in Dujardin 1841) |  | | 10 |  |  | In Dujardin (1841): - Cells are irregularly ovoid.  - The remarkable large vacuole in the cell.  - One flagellum  - Crawled at the bottom like amoeba or swim with changing the shape. |
| *Monas gibbosa* Dujardin | The images did not publised. |  | | 10 |  |  | In Dujardin (1841):  - Cells are oblong, angular, irregularly swollen and hunchbacked. Some were narrowed posterior and others were narrowed both end. |
| *Monas gliscens* Ehrenberg | 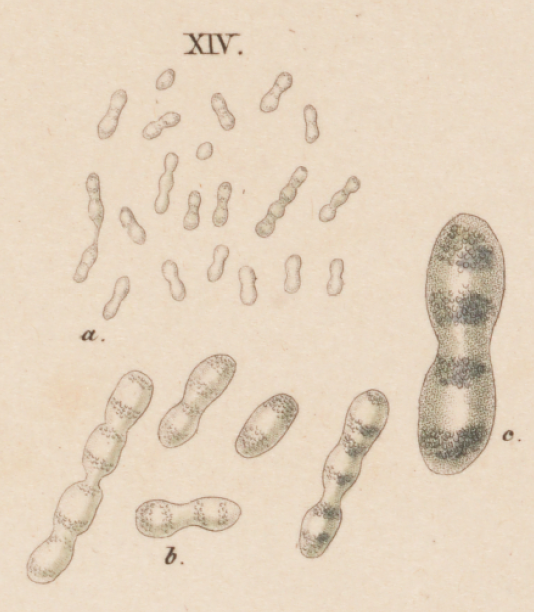  (Fig. 14. *M. gliscens.* a. represents a group of 20 animals in various degrees of self-division and also individual simple ones with an enlargement of 290 times in diameter, b. 5 animals are enlarged 820 times. One of these is simple, the others form rod-like structured monads - sticks - by simple or multiple queer division without complete separation of the parts. Two of them are in the process of simply dividing, hence doubly; in the state of the first division, before it was completed, began to divide anew in one of the parts; thus 3 connected individuals were created. Finally, one thing, understood in the first division, began new self-division in both parts before its completion; in this way 4 still connected animals or a movable rod were created, c. is an animal in the process of queer division with a 2000 times increase in diameter. Plate 1 in Ehrenberg 1838) |  | | 5.2 |  |  | In Ehrenberg (1838):  - Cells are oval, rounded at both end.  - Cells are continually gliding.  - One cell is often divided to four cells.  - The form of self-division is similar to bacterium. |
| *Monas globulus* Dujardin | 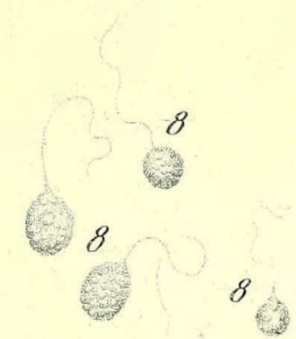  (Plate 4, Fig. 8. in Dujardin 1841) |  | | 9-14 |  |  | In Dujardin (1841):  - Cells are globular, almost constant in shape.  - One flagellum  - Marine |
| *Monas grandis* Ehrenberg | 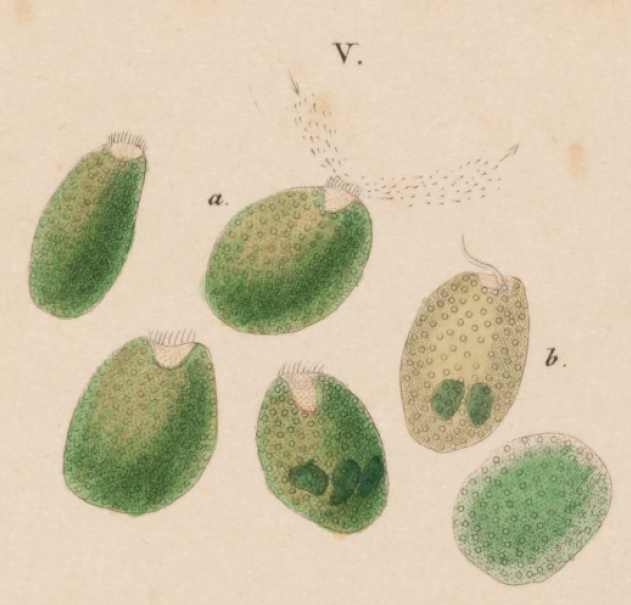  (Fig. 5. *M. grandis.* 6 individuals are shown in different positions, 5 of which were drawn in 1832, 1 in 1835; all are diametrically enlarged 290 times. Fig. A. is one of the individuals observed in 1832 swirling. The multitude of eyelashes around the mouth of this and the other may owe their origin to the optical illusion mentioned. Fig. B. is a drawing from 1835. The two oval bodies in the abdomen may be testicles. Plate 1 in Ehrenberg 1838) | 55.5 | |  |  |  | In Ehrenberg (1838):  -Cells are oval, rounded at both ends.  - Cells are green because of the small green round granules in the cell body.  - Cells periodically have colorless form.  - Cells having cilia and very short thread-like trunk (length in 0.25 to 0.33 of the cell body).  - Having diaphanous mouth. |
| *Monas hyalina* Ehrenberg | 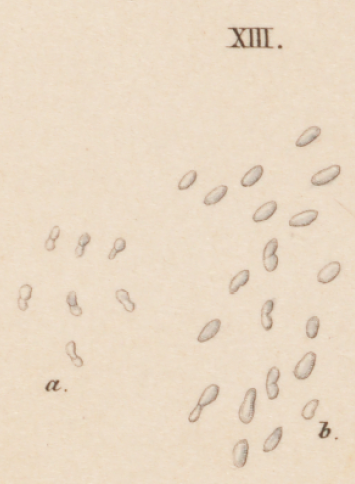  (Fig. 13. *M. hyalina.* a. there are 7 individuals in the process of queer division of the double 0.002 line large forms observed in Tobolsk in the stagnant water of the Tobol, magnified 525 times, b. are 20 individuals from the Neva water in St. Petersburg, from 1/240 to 1/500 line size. The constricted or doubled individuals are in the queer division. Plate 1 in Ehrenberg 1838) |  | | 4.0-8.3 |  |  | In Ehrenberg (1838):  - Cells are oval, rounded at two ends and colorless.  - The cell size and movement are similar to *Bodo didymus* and *Bodo saltans*. |
| *Monas inanis* Ehrenberg | 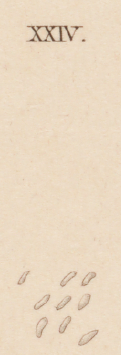  (Fig. 24. *Monas inanis.* They present the 9 individuals of the *Monas inanis* drawn in Libya in 1820 and enlarged 200 times. Some are conceived from the narrow side, others from the broad side. Plate 1 in Ehrenberg 1838) |  | | 6.6 |  |  | In Ehrenberg (1838):  - Shape of the cell is tapered to one side and slightly compressed laterally.  - Cells have wavering movement. |
| *Monas intestinalis* Dujardin | The images did not publised. |  | | 17 |  |  | In Dujardin (1841):  - Cells have elongated body and continuously varying in shape rounded at posterior and gradually narrowed to anterior.  - Ripple movement all of the cell body.  - It guessed as a *Bodo* species. |
| *Mons kolposa* Ehrenberg | 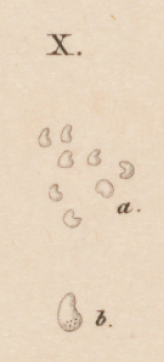  (Fig. 10. *M. kolpoda.* a. are 8 individuals swarming around each other in a droplet close to complete evaporation, magnified 525 times, b. is a single individual after enlarging the diameter 800 times. Plate 1 in Ehrenberg 1838) | Under 3.3 µm | | |  |  | In Ehrenberg (1838):  - Cells are shape of a kidney.  - Shape of the cell is tapered to anterior.  - Cell have wavering movement. |
| *Monas lamellulia* Müller | 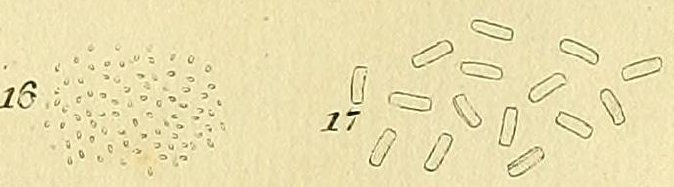  (Fig. 16. *M. lamellulia*, enlarged. Fig. 17. More enlarged. Plate 1 in Müller 1786) |  | |  |  |  | In Müller (1786):  - Cells are very thin and colorless or white.  - Cell swim slowly. |
| *Monas lens* Müller | 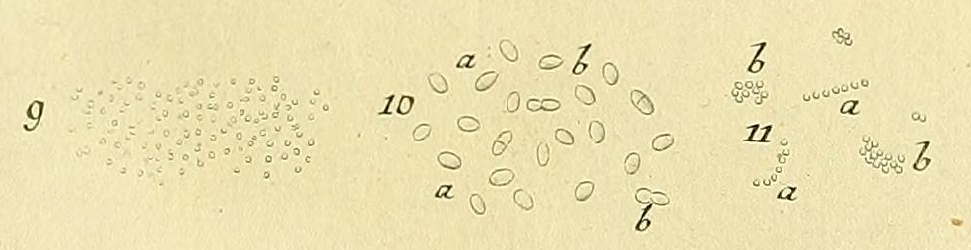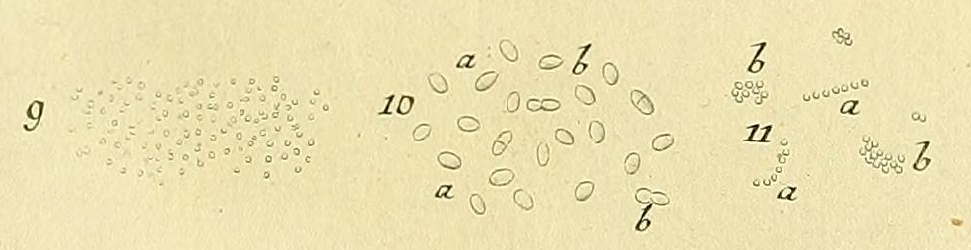  (Fig. 9. M. lens, enlarged. Fig. 10. More enlarged, a. single cell, b. cell division. Fig. 11. a. Cell forms a row, b. Cells grouped together. Plate 1 in Müller 1786)  **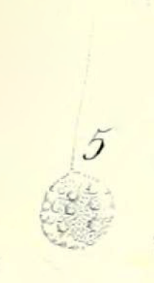**  (Plate 3, Fig. 5 in Dujardin 1841)  **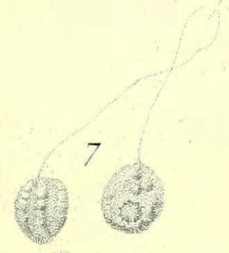**  (Plate 4, Fig. 7 in Dujardin 1841) | 13-17 in diameter | | | 3-5 times longer than cell body |  | In Müller (1773):  - Shape of the cell is intermediate between oval and spherical.  - Cells do not have any intestine.  In Dujardin (1841):  - Cells are globule.  - One flagellum. |
| *Monas lindahlii* | 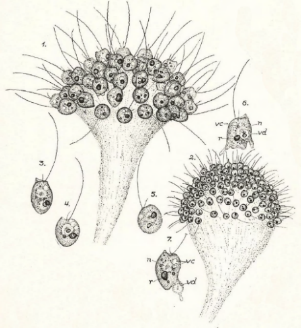  (Fig. 1-7. *M. lindahlii* n. sp. A moderately large colony with the compound gauntlet foot more enlarged, 670 x, 2. A larger colony, less enlarged, 340 x, 3-7; free-floating single cells, vc; pulsating vacuoles, vd; digestion vacuole with food, in Fig. 7 the digestive vacuole with food residues is led to the rear end and will soon become indigestible contents, r; a strongly refractive grain in the protoplast, n; nucleus, 6; with lobopodia. Plate LVII in Skuja 1956) | 4-9 | | 7-12 | 3 times longer than cell body. | 0.16-0.33 times of the cell body. | In Skuja (1956):  - Cells are rounded ovate, rounded obovate and make mushroom shaped colony.  - The colony is 150 µm in width, 300 µm in height and connected using gelatinous stalk.  - Two unequal flagella  - Consume bacteria, small green algae and chrysophycean algae. |
| *Monas longicilia* Dangeard | 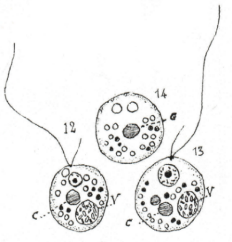  (Fig. 12-14 *M. longicilia* n. sp. feeding on bacteria and having, apart from the nourishing vacuoles, one or two chromies c in the cytoplasm: inside this there are spherules, many of which are colored with cresyl blue, while the aufres remain colorless. Plate XXXVIII in Dangeard 1934) | 10-16 in diameter | | | 3-4 times longer than cell body |  | In Dangeard (1934):  - Cells are spherical.  - Two unequal flagella with strikingly long one. |
| *Monas mediovaculoata* Skuja |   (Plate III in Skuja 1939; Images captured from Grossmann et al. 2016) | 6-16 | | 16-20 | Equal or little longer than cell length. | 0.16 times of the cell length. | In Skuja (1939):  - Cell are almost spherical and sometimes oval, rounded, cylindrical, pyriform.  - Two unequal flagella.  - Consume the unicellular algae and bacteria as a prey.  - Short line of mouth usually be seen in anterior. |
| *Monas mediovaculoata* var, *facilis* n. var. | *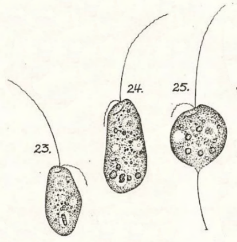*  (Fig. 23-25. *M. mediovacuolata* Skuja var. *facilis* n. var. 23 and 24; free-swimming, 25; fixed. Plate LVI in Skuja 1956) | 5-12 | | 6-16 | 20 | 4-6 | In Skuja(1956):  - Cells are cylindrical in floating state, almost spherical to rounded-ovoid in fixed state.  - Two unequal flagella.  - Consume small green algae, diatom and bacteria as a prey.  - Forms a long plasma foot (stalk).  - Short line of mouth usually be seen in anterior. |
| *Monas mica* Müller | *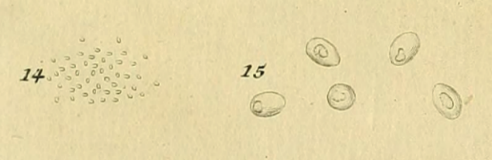*  (Fig. 14. *M. mica*, enlarged. Fig. 15. More enlarged. Plate 1 in Müller 1786)  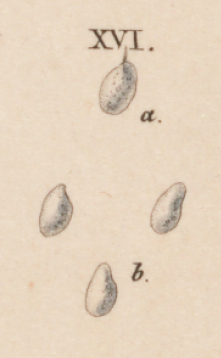  (Fig. 10. *M. mica.* Fig. a. is the shape drawn by me in Buchtarma from the water of the Irtysh. It measured 1/120 line and is enlarged 245 times. The fine points inside may well be filled stomach cells. Fig. b. 6 are 3 forms observed in Berlin. A trace of the large central seminal gland, however, was probably also noticeable. See *Chilomonas*. Plate 1 in Ehrenberg 1838) |  | |  |  |  | In Müller (1773; 1786):  - Cells are oval or spherical.  - Oval shaped structure is in the cell. It is motile and located either in the center or anterior or posterior of the cell.  In Ehrenberg (1838):  - Cells are oval (16.6-20.0 µm).  - Cells have swirling and wobbling movement. |
| *Mons mior* var. *praegnans* n. var. | 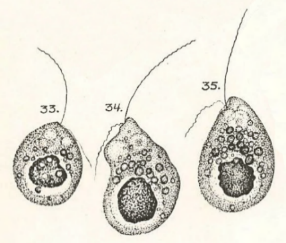  (Fig. 33-35. *M. maior* var. *praegnans* n. var.) | 10-15 | | 13-20 | Almost equal to cell length | 0.33-0.5 times of the cell length | In Skuja(1956):  - Cells are oval, tapering apex more than rounded.  - Two unequal flagella.  - Large digestive vacuole in the posterior of the cell. |
| *Monas neglecta* Skuja | *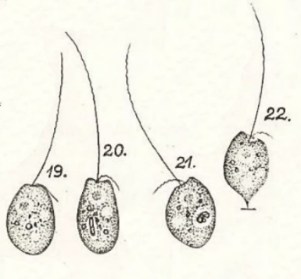*  (Fig. 19-22. *M. neglecta* n. sp. Plate LVI in Skuja 1956) | 4-8 | | 7-11 | 2-3 times longer than cell length. | 0.5-0.7 times of the cell length. | In Skuja (1956):  - Cells are ovate, cylindrical to ellipsoidal with obliquely truncated anterior.  - Two unequal flagella.  - Forms plasmatic short stalk. |
| *Monas nodasa* | 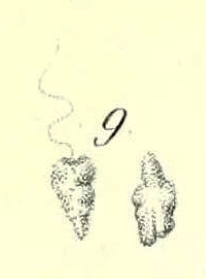  (Plate 4 Fig. 9 in Dujardin 1841) |  | | 11.3 |  |  | In Dujardin (1841):  - Cells are oblong, knotty with narrowed posterior.  - One flagellum.  - Marine |
| *Monas oblonga* Dujardin | The images did not published |  | | 7.4-16.4 | 11.8-16.4 |  | In Dujardin (1841):  - Cells are ovoid, oblong. |
| *Monas ocellus* Müller | 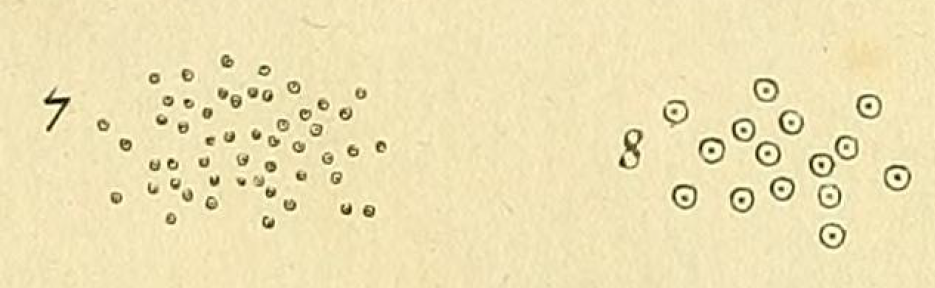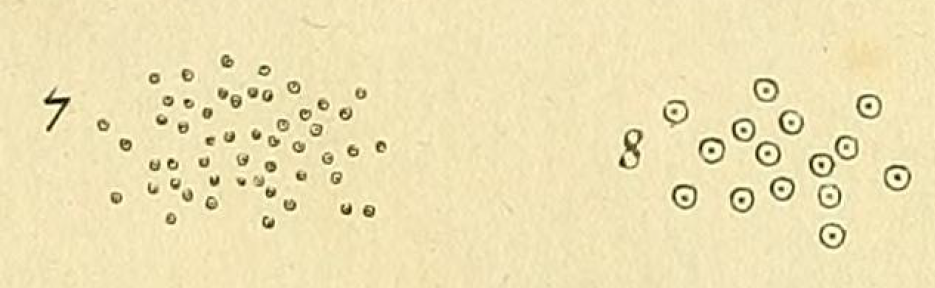  (Fig. 7. *M. ocellus*, enlarged. Fig. 8. More enlarged. Plate 1 in Müller 1786) |  | |  |  |  | In Müller (1786):  - Cell have black dot in middle of the cell. |
| *Monas ochracea* Ehrenberg | 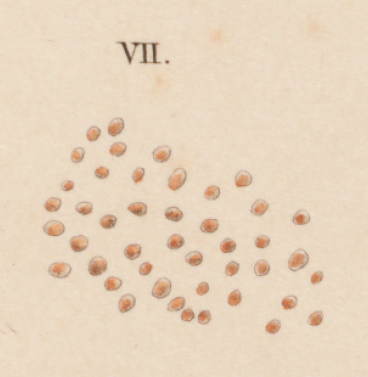  (Fig. 6. *M. ochracea.* A bunch of 40 and some individulas magnified 290 times. The largest are 1/500 of a line. Plate 1 in Ehrenberg 1838) | Under 4 µm | | |  |  | In Ehrenberg (1838):  - Cells are globular and have red-yellow (ocher) color.  - |
| *Monas okenii* Ehrenberg | The images did not publised. |  | |  |  |  | In Ehrenberg (1838):  - Cells are cylindrical, somewhat curved, colorless but some individuals have a red or green color.  - Having flagellum (half of the cell body).  - Cells have swaying movement |
| *Monas ovalis* Ehrenberg | 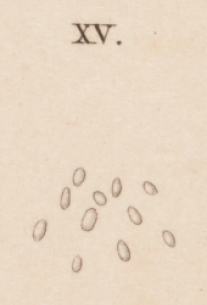  (Fig. 15. *M. ovalis.* There are 525 times magnified 11 individuals of the *M. ovalis* from the *Anodonta* of Ob. Plate 1 in Ehrenberg 1838) | 2.5 | |  |  |  | In Ehrenberg (1838):  - Cells are oval, rounded at both ends.  - Cells have trembling movement. |
| *Monas profunda* Skuja | 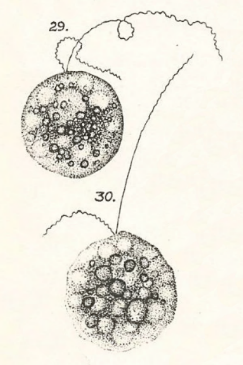  (Fig. 29-30. *M. profunda* n. sp. Plate LVI in Skuja 1956) | 13-20 in diameter | | | Longer than cell body |  | In Skuja (1956):  - Cells are globose.  -Having coarse-grained plasma (cell outlines appear clearly and irregularly wavy).  - Consume the small green algae as a prey.  - The color of food vacuole is often changed in different stages of digestion (green to brown and yellowish). |
| *Monas pulvisculus* Müller | 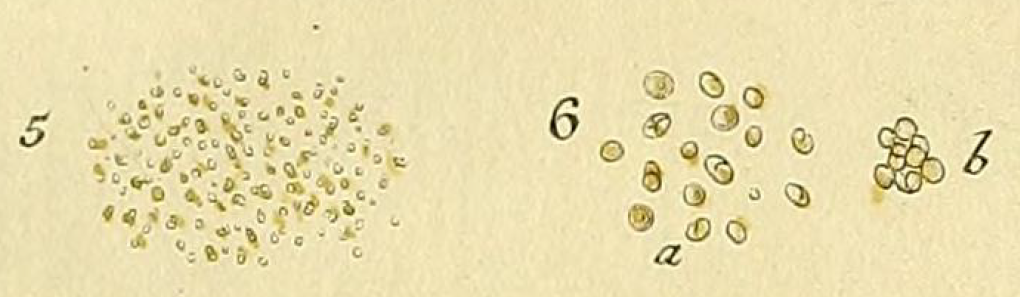  (Fig. 5. *M. pulvisculus*, enlarged. Fig. 6. More enlarged. a. curved line on the cell surface, b. cells grouped together. Plate 1 in Müller 1786) |  | |  |  |  | In Müller (1786):  - Cells are spherical with greenish margin.  - Curved line in the cell surface is formed before cell division. |
| *Monas punctum* Müller | 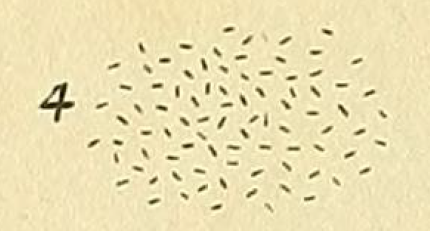  (Fig. 4. *M. punctum*. Plate 1 in Müller 1786) |  | |  |  |  | In Müller (1786):  - Cells are very small and black.  - The width of cell is considerably longer than length. |
| *Monas punctum* Ehrenberg | 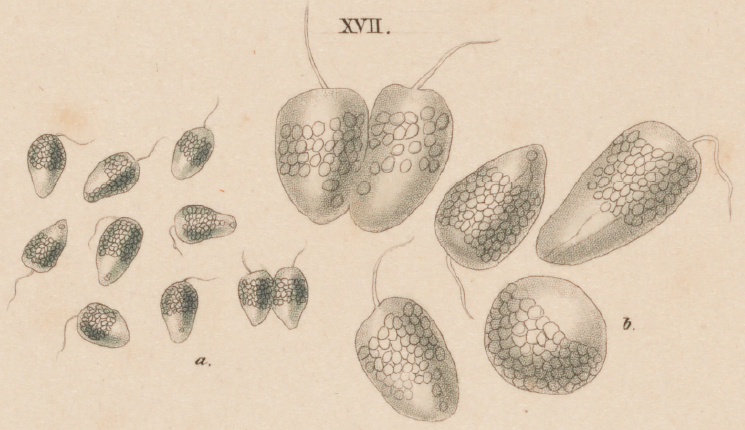  (Fig. 17. *M. punctum.* a. 9 to about 1/96 line large individuals of the Monas Punctum of Berlin, 290 times enlarged in diameter. One of them is in the longitudinal division. b. are 5 of the same, enlarged 530 times. In two of these forms the rear light spot is divided, one is far advanced in the complete longitudinal division of the body, and one has contracted spherically. Plate 1 in Ehrenberg 1838) |  | | 20.8 |  |  | In Ehrenberg (1838):  - Cells are oval, truncated anterior, thinned posterior.  - Having flagellum (length in half of the cell body).  - The wide belt of internal granule in the middle of the cell. |
| *Monas scintillans* Ehrenberg | 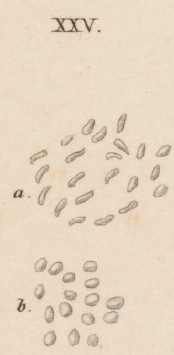  (Fig. 25. *Monas scintillans.* The two groups shown are one living and one dead. Fig. a. are 22 living individuals from Berlin, whose largest line reaches 1/384, enlarged 380 times. Fig. b. 16 dead, therefore disc-shaped individuals are from there. Plate 1 in Ehrenberg 1838) | 0.3-0.5 of the cell length | | 4-5.2 |  |  | In Ehrenberg (1838):  - Shape of the cell is tapered to one side and somewhat compressed.  - Cells have brisk, wobbling movement |
| *Monas simplex* Ehrenberg | 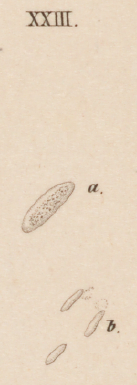  (Fig. 22. *Monas simplex.* Fig. a. is an individual observed near Berlin in 1827, 1/144 line in size, enlarged 345 times. Fig. b. are 3 specimens observed in Egypt from Lachen near Bulak not far from Cahira. They were 1/300 line long and magnified 200 times. Plate 1 in Ehrenberg 1838) |  | |  |  |  | In Ehrenberg (1838):  - Cells are cylindrical and have a line on the surface.  - Cells have sliding and rotary motion. |
| *Monas socilais* Ehrenberg | 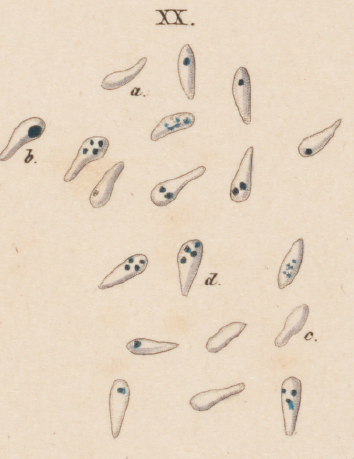  (Fig. 20. *Monas socialis.* In all, nineteen individuals nourished with indigo are shown in such a grouping as is often seen. The various prevailing individual forms and positions are taken into account. Fig. a. has not absorbed any color and bends when swimming. Fig. b. is turned back with the front part and has only 1 stomach cell, but it is very strongly filled. Fig. c. is preparing for queer division and has not consumed any indigo. Fig. d. has filled four stomach cells, the pointed end of the anterior part is everywhere. They swim in different directions. Plate 1 in Ehrenberg 1838) | 0.5 of the cell length | | 14.0 |  |  | In Ehrenberg (1838):  - Cells are oblong, conical.  - Form colony using stalk in posterior side. |
| *Monas tingens* Ehrenberg | 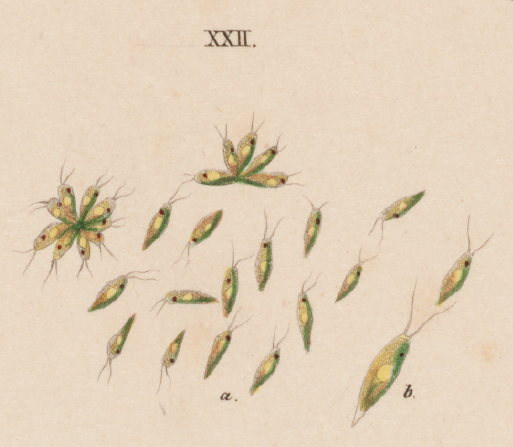  (Fig. 22. *Monas tingens.* Plate 1 in Ehrenberg 1838) | 0.25-0.3 of the cell length | | 6.6-13.8 |  |  | In Ehrenberg (1838):  - Cells are green and forms colony.  - Having eye spot. |
| *Monas tranquilla* Müller | 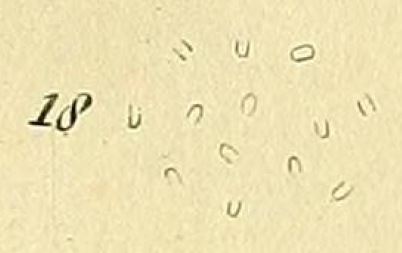  (Fig. 18. *Monas tingens.* Plate 1 in Müller 1786) |  | |  |  |  | In Müller (1786):  - Cells are oval and colorless. |
| *Monas umbra* Ehrenberg | 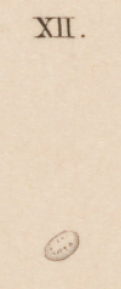  (Fig. 12. *Monas umbra.* The individual is enlarged 525 times in diameter. Inside, traces of organs are can be shifted, which then were not understood as clearly as they are now. Plate 1 in Ehrenberg 1838) | 10 | |  |  |  | In Ehrenberg (1838):  - Cells are oval, rounded at both end. |
| *Monas uniguttata* Skuja |   (Plate Ⅲ 17 in Skuja 1939; Images captured from Grossmann et al. 2016) | 4-6 | | 7-12 | 2-3 times longer than cell cell length. | 0.3-0.5 time longer than cell length | In Skuja (1939):  - Cells are obovate, ovate or cylindrical.  - Two unequal flagella.  - Having amoeboid stage.  - Having stalk, 1.5 times longer than cell body. |
| *Monas uva* Müller | 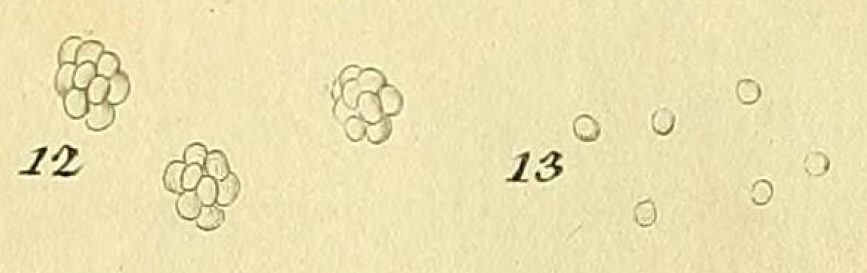  (Fig. 12. *M. uva.* Fig. 13. Globous single cell, enlarged. Plate 1 in Müller 1786) |  | |  |  |  | In Müller (1786):  - Cells grouped together.  - Cells are globulose. |
| *Monas varians* Dujardin | The images did not publised | 40-52 | | |  |  | In Dujardin (1841):  - Cells are oblong, anterior is narrower and constantly changing in shape.  - The continual change is similar to *Peranema,* but do not having integument. |
| *Monas vinosa* Ehrenberg | 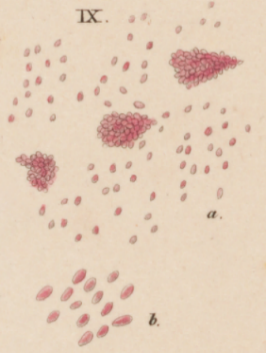  (Fig. 6. *M. vinosa.* a. are a few hundred individuals with a 450-fold increase in diameter. Some are heaped together and quiet, probably dead, about 70 swarming around slowly, trembling, b. shows 14 little more animals, namely 820 times enlarged. Plate 1 in Ehrenberg 1838) | 2-4 | | |  |  | In Ehrenberg (1838):  - Cells are ovoid and have vinous (wine-red) color.  - Cells have quivering and very slow movement. |
| *Monas vorax* Dangeard | 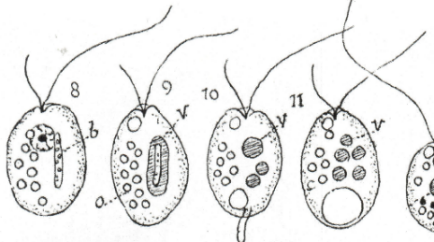  (Fig. 8-11 *M. vorax* n. sp. – with ingested filamentous bacteria, the chromidia in the cytoplasm and the presence of leucosine in several individuals. Plate XXXVIII in Dangeard 1934) |  | | 15 |  |  | In Dangeard (1934):  - Cells are elliptic.  - Two unequal flagella. |
| *Monas vorax* Skuja |   (Plate Ⅲ 17 in Skuja 1939; Images captured from Grossmann et al. 2016) | 10-13 | | 13-16 | 2-2.5 times longer than cell length. | Half of the cell length. | In Skuja (1939):  - Cells are obovate or ovate, rounded anterior with tapered posterior (13-16 µm in length, 10-13 µm in width).  - Two unequal flagella (Long: 2-2.5 times longer than cell body, Short: half of the cell body)  - Consume the bacteria and algae as a prey. |
| **Genus *Pedospumella*** | | | | | | | |
| *Pedospumella elongata* (Stokes) Boenigk et Grossmann |   (Fig. 1. Diagrammatic longitudinal section of *Spumella elongata.* F_1_, F_2_, long and short flagella; MT,  microtubular root; B, epiphytic bacterium; G, Golgi body; C, t. s. crystal; CV, contractile vacuole;  H, vesicle in ER containing flagella hairs; L, area containing presumed leucosin; FV, food vacuole;  M, mitochondrion·; LP, leucoplast; BR, banded root. in Belcher and Swale 1976) |  | |  |  |  | In Stokes (1886):  - Cells are elongate-ovate (11.3 µm in length) and changeable in shape, posteriorly developed pedicel, the lip usually prominent.  - Two unequal flagella (Long flagellum is sub-equal to the cell length, short flagellum is one-third that length).  In Belcher and Swale (1976):  - Motile cells are oval (8-10 µm in length, 6-8 µm in width), narrowing at the anterior end.  - Two unequal flagella (Length of long flagellum is equal to cell length and length short flagellum is less than half this length). |
| *Pedospumella encystans* Boenigk et Grossmann |   (Fig. 4b. strain JBM/S11 in Findenig et al. 2010)    (Fig. 5f. strain *Pedospumella encystans* in Findenig et al. 2010)    (Fig. 1B. strain 1006 = *Pedospumella encystans* in Grossmann et al. 2016) |  | |  |  |  | In Findenig et al. (2010):  - Vegetative cells usually spherical, sometimes elongated or posteriorly pointed (3.0-6.2 µm in diameter).  - Two unequal flagella (Long: 2 to 3.5 times as long as the cell body, Short: 0.7-1 times as long as the cell body).  - Stomatocysts are spherical (6.6-10 µm in diameter) and have regular pore with a concave pseudoannulus. |
| *Pedospumella sinomuralis* Boenigk et Grossmann |   (Fig. 1I. strain JBCS23= *Pedospumella sinomuralis* n. sp. in Grossmann et al. 2016) | 1.9-5.6 in diameter | | | Up to 9.5 |  | In Grossmann et al. (2016):  - Cells are mostly spherical, sometimes elongated or posteriorly pointed.  - Two unequal flagella.  - Long flagellum have tripartite mstigonemes. |
| **Genus *Poteriospumella*** | | | | | | | |
| *Poteriospumella lacustris* Boenigk et Grossmann |   (Fig. 5e. *Poteriospumella lacustris* in Findenig et al. 2010) | 3.0-5.0 in diameter | | | 1.5 to 2 times as long as the cell body | 0.5 times as long as the cell body | In Findenig et al. (2010):  - Cell usually spherical, sometimes elongated or posteriorly pointed.  - Two unequal flagella. |
| **Genus *Oikomonas*** | | | | | | | |
| *Oikomonas mutabilis* | 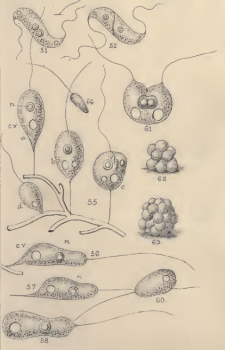  (Fig. 55-64. *Oikomonas mutabilis*. 55, A group of four monads attached to vegetable fibre, showing at a and b normal sedentary forms, at c an example ingesting  food-matter at its lateral periphery, and at d a young and recently adherent example, not having yet developed a filiform pedicle, x 800 ; 56, an adult monad about to exchange its sedentary for a free-swimming condition ;  57, the same monad detached and free-swimming, still retaining an attenuation of its posterior and previously fixed extremity ; 58, typical free-swimming zooid ; 59 and 60, more aberrant forms ; 61, a motile zooid dividing by longitudinal fission ; 62 and 63, spore-masses produced by segmentation of encysted animalcules ; 64, a young monad developed from a spore. Plate ⅩⅠⅠⅠ in Kent 1880) | 16.9 to 33.8 | | |  |  | In Kent (1880):  - Cells are changeable, spherical , ovate, symmetrically ovate, pyriform or subspherical.  - Having peciel, equal to body in length. |
| **Genus *Segregatospumella*** | | | | | | | |
| *Segregatospumella draxosaxi* Boenigk et Grossmann |   (Fig. 1C. strain AR3A3= *Segregatospumella dracosaxi* n. gen. n. sp. in Grossmann et al. 2016) | 1.2-5.6 in diameter | | | Up to 8 |  | In Grossmann et al. (2016):  - Cells mostly spherical, sometimes elongated or posteriorly pointed.  - Two unequal flagella.  - Long flagellum have tripartite mastigonemes. |
| **Genus *Spumella*** | | | | | | | |
| *Spumella beauchampii* (Hovasse) Silva | 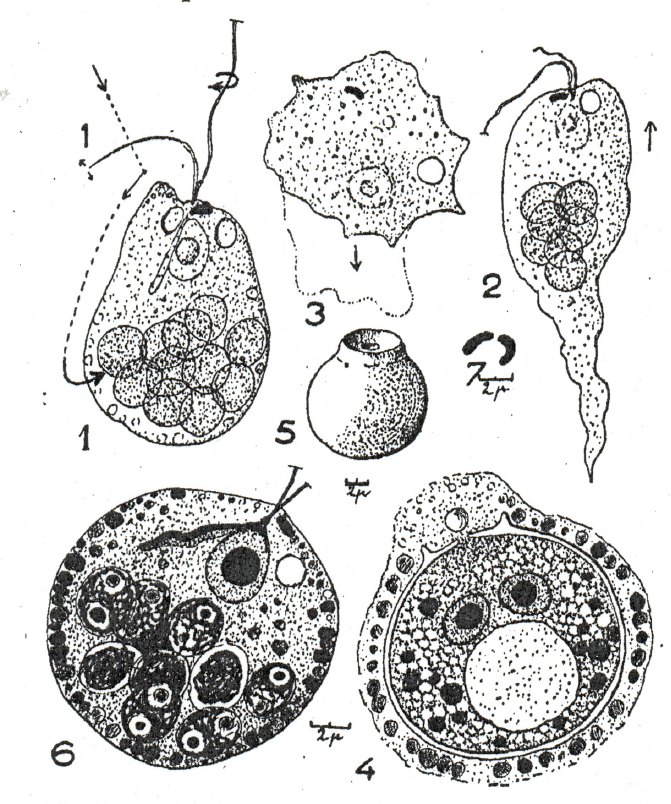  1. *Oicomonas beauchampi* n. sp. *In vivo*, in position of capture of proles. The arrows indicate the path of these and the beating of the flagella. Nucleus, parabasal, pulsatile vacuoles and stigma. - 2, Id., Crawling on the substratum, flagella preserved. Nucleus, vacuole, stigma. - 3, Id. Amoeboiod phase: nucleus, pulsatile vacuole, stigma. - 4, Champy fixation and cut, Formation of the cyst and its plug. - 5, Isolated cyst, without plug. - 6, Champy binding. Chopped off. Nucleus and karyosome, mastigosome and parabasal, nucleus cone, pulsatile vacuole, stigma, Chlorella in digestion. Free inclusions. - 7, Detail of a stigma, double type. | Less wide than cell length | | 11-13. | 30 | 10 | In Hovasse (1943) :  - Cells are ovoid.  - Two unequal flagella.  - Having stigma  - Crawling behavior  - Chlorella in food vacuole  - Stomatocyst is spherical, collar and planar annulus around the pore |
| *Spumella bureschii* (Valkanov) Boenigk et Grossmann | 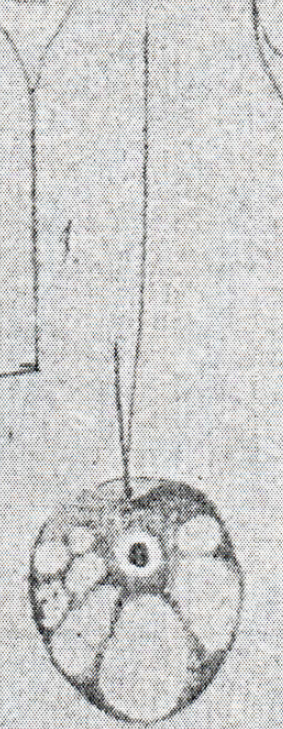  (Fig. 18. *M. bureschii* n. sp. Plate 2 in Valkanov 1926)    (Fig. 4d. strain JBL14 in Findenig et al. 2010)    (Fig. 1J. strain JBL14 = *S. bureschii* nov. comb. in Grossmann et al. 2016) |  | |  |  |  | In Valkanov (1926):  - Cell are spherical to ovoid (6 µm in length).  - Two unequal flagella (Long: 1.5 times longer than cell body, Short: 0.5 times longer than cell body).  - The vacuole and nucleus located in anterior part of the cell.  In Findenig et al. (2010)  - Stomatocysts were spherical to slightly oval (5.17-8.09 µm in diameter)  - Broad conical collar which outer margin is continuous with stomatocyst body.  In Grossmann et al. (2016)  - Cells are mostly spherical, sometimes elongated or posteriorly pointed (2.9-7.4 µm in diameter).  - Two unequal flagella (Long: up to 14.2 µm, Short: Did not described.). |
| *Spumella dinobryonis* (Skuja) Zhukov | 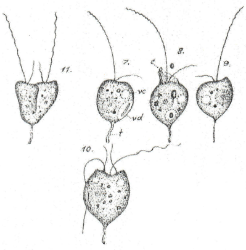  (Fig. 7-11. *M. dinobryonis* n. sp. 7 with a bacterium in the digestive vacuole (vd): 8 when the body of food is ingested, a plasma cup (c) forms temporarily at the front end, into which the food is curled up by the vibrations of the main flagella; 10. 11 in the longitudinal division: *t* plasmatic stalk with which the monad attaches itself to a substrate. Plate XXXV in Skuja 1948) | 5-11 | | 5-13 |  |  | In Skuja (1948):  - Cells are usually rounded but changeable.  - Two unequal flagella.  - Food vacuole is variously colored. |
| *Spumella gregaria* Tanichev | 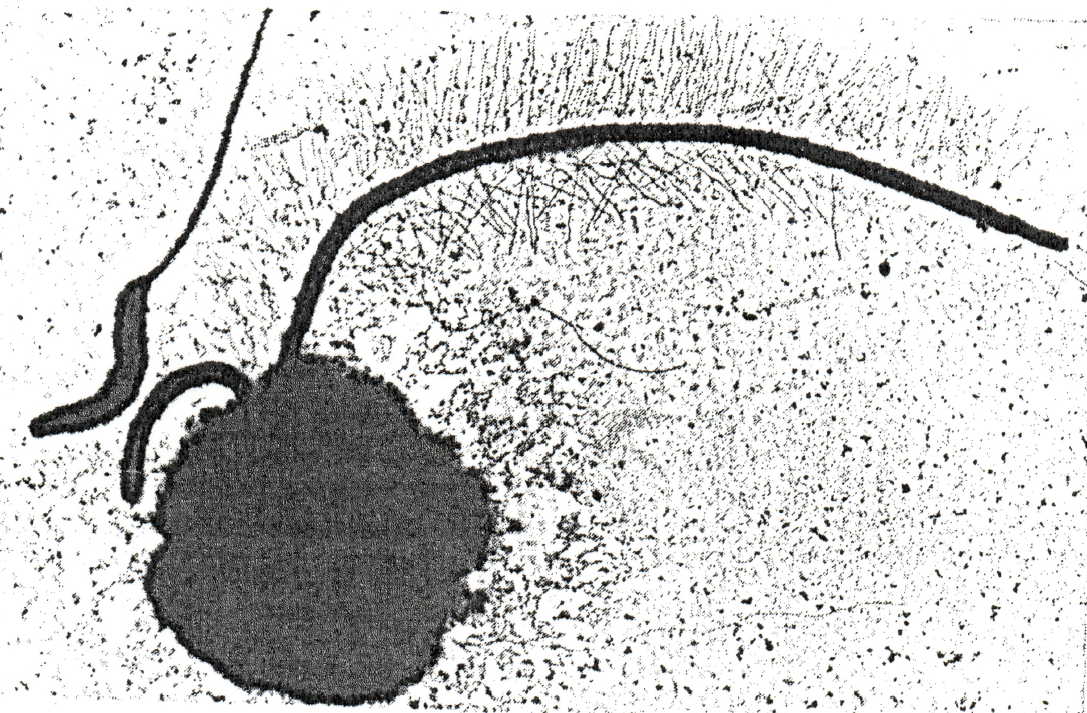  (Fig. 4. *S. gregaria* vegetative cell in Tanichev 1993)  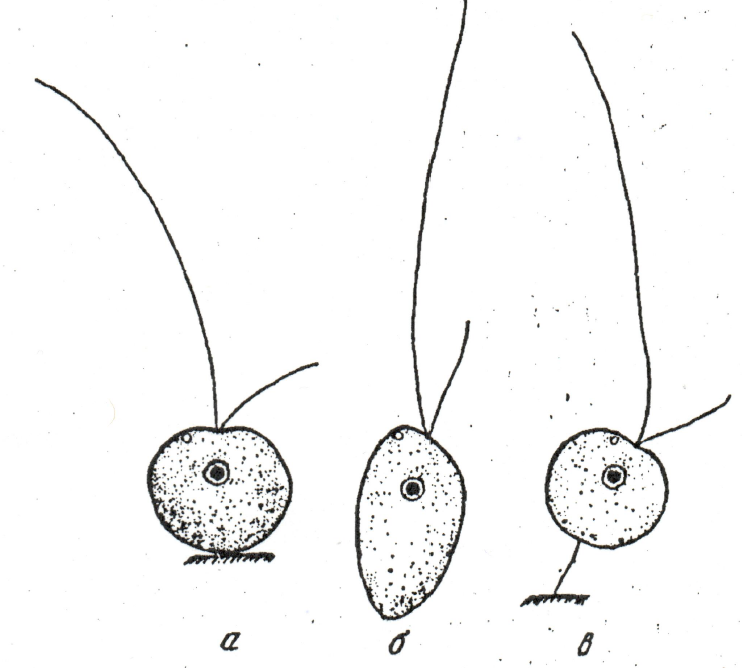  (Fig. 5. Variation in the shape of the *S. gregaria* cell body; a, в-attached cells; б-free floating stage. in Tanichev 1993)  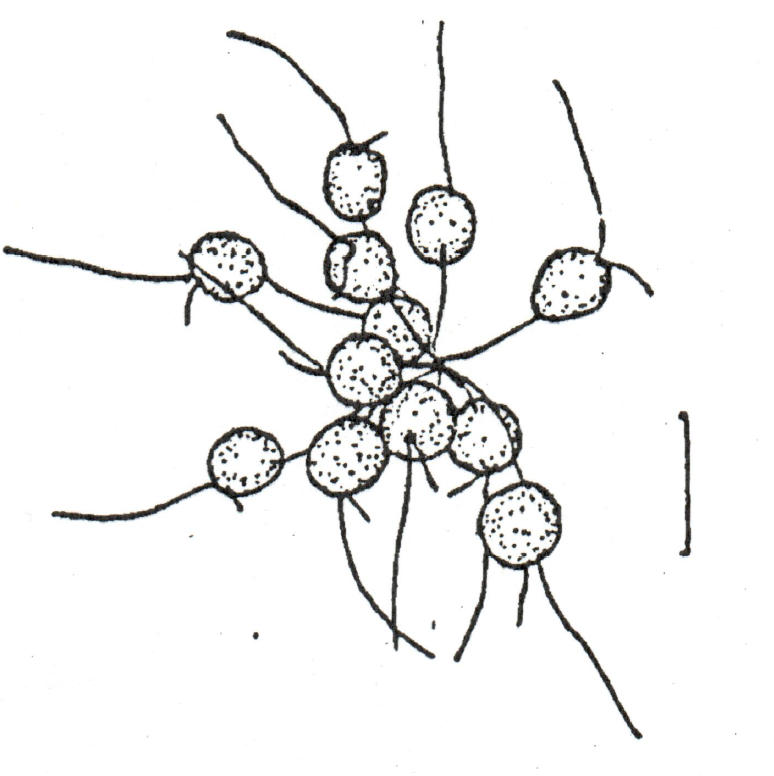  (Fig. 7. *S. gregaria*, free floating coloy. in Tanichev 1993) | 3.7-5.5 | | 3.7-7.5 | 2.5-3.5 times longer than cell cell body | 0.7-1 of the cell body. | In Tanichev (1993)  - Cells are round or oval in swimming individuals.  - Two unequal flagella.  - Attached to substratum with pedicel and individuals unite free floating colony using pedicel. |
| *Spumella guttula* (Ehrenberg) Kent | 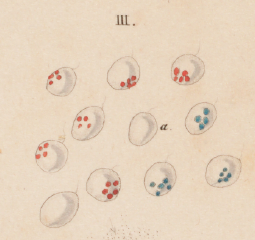  (Figure 3. *Monas guttula*. Fig. A. is an empty (hungry) individual, surrounded by colored nourished, at 450 times diametrical magnification. Plate 1 in Ehrenberg 1838.)  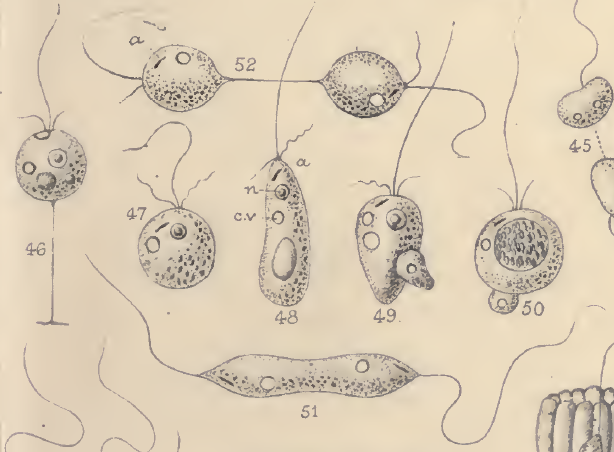  (Fig. 46-52. normal attached monad;  47 and 48, free-swimming monads; 49 and 50, illustrating conjugative process of larger and smaller monads; 51 and 52, successive phases of longitudinal  fission. Plate XIV in Kent 1881)  ***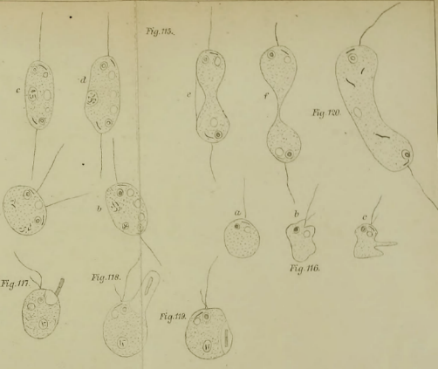***  (Fig. 115 a-f. Successive stages of longitudinal division. Fig. 116 a-c. Partial pieces, in amoeboid motion.  Fig. 117-119. Formation and migration of the nourishing vacuole. Fig. 120. Stage of division of a giant specimen.) |  | | |  |  | In Ehrenberg (1838):  - Cells are strikingly big and colorless  - Several bubbles in the cell.  - Cells have flagellum  In Kent (1880/1):  - Cells are globose (10.16 µm in diameter), ovate, pyriform or elongate  - Three flagella (One long, two short)  - Attached to substratum with pedicel.  In Fisch (1885):  - Cells are spherical, egg-shaped.  - Young cells showed amoeboid stage. (Extrude the pseudopodia).  - Having line of the mouth. |
| *Spumella hovassei* (Fiatte & Joyon) Bourrelly | 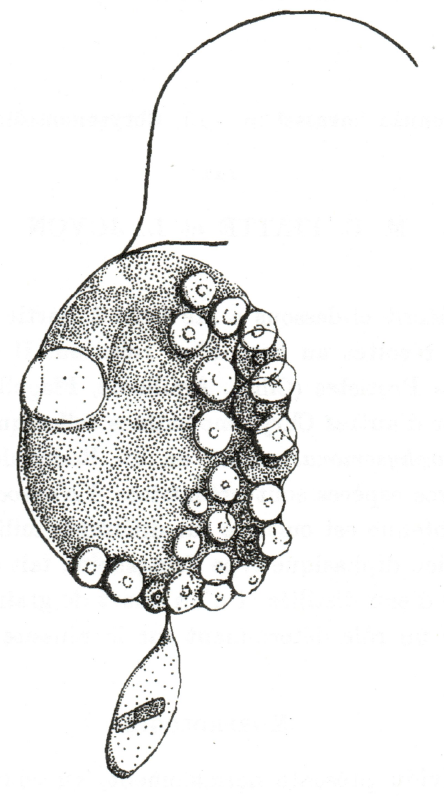  (Fig. 1. *H. hovassei*, *in vivo* aspect. The two anterior flagella are very unequal. On the side is a pulsatile vacuole; In contrast, there is a field of mucliferous bodies. In the posterior region, there is emission of a cytoplasmic veil and capture of a bacterium. in Fiatte and Joyon 1965)  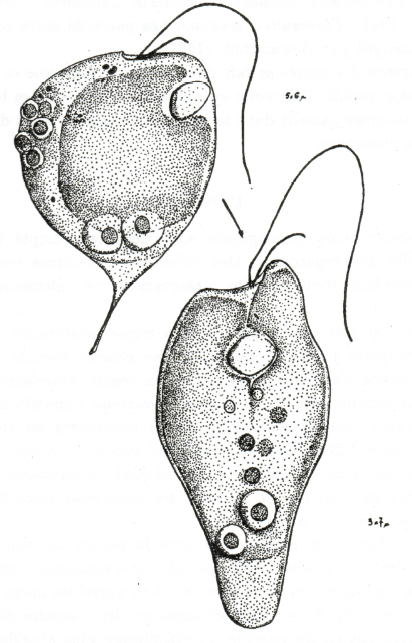  (Fig. 2. *H. hovassei*, different *in vivo* aspects of the same highly metabolic cell. Note in this individual the volumetric importance of the central leucosine vacuole. in Fiatte and Joyon 1965)  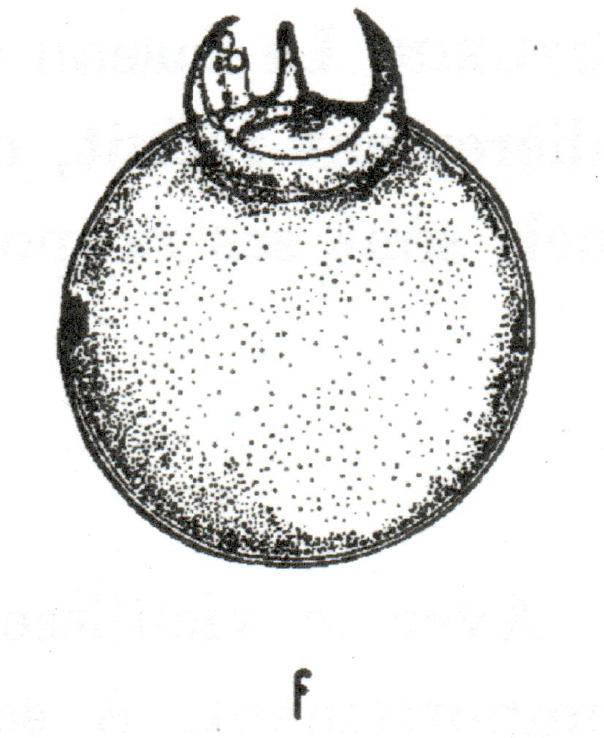  (Fig. 3. *H. hovassei*, Formation of cyst. in Fiatte and Joyon 1965) | 5-8 in diameter | | | 12-15 | 3-5 | In Fiatte and Joyon (1965):  - Cells are roughly spherical, usually truncated anterior with small apical depression. Posterior part often tapered  - Two unequal flagella.  - Having large number of shiny granules on the cell surface. (It is considered muciferous body of *Ochromonas hovassei*, *Cyclonexis annularis*, *Chromulina erkensis*).  - Stomatocyst is 7-8 µm in diameter.  - Stomaatocyst have three tap-like collar around the pore and taps are slightly curved inward. |
| *Spumella lacusvadosi* Boenigk et Grossmann |   (Fig. 1N. JBNZ39 = *S. lacusvadosi* n. sp. in Grossmann et al. 2016) | 1.5-7.1 in diameter | | | 10.4 | Did not described. | In Grossmann et al. (2016)  - Cells are mostly spherical, elongated or posteriorly pointed shape.  - Two unequal flagellates. |
| *Spumella mior* (Skuja) Zhukov | 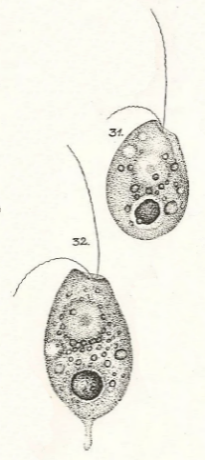 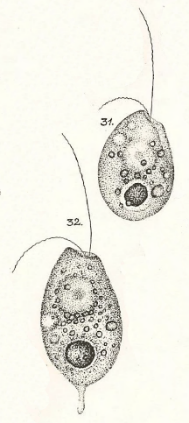  (Fig. 31-32. *M. maior* n. sp. Plate LVI in Skuja 1956) | 18-25 | | 25-40 | Equal or slightly longer than cell body | Half or slightly longer than half of the cell body. | In Skuja (1956)  - Cells are ovate.  - Two unequal flagella.  -Multiple pseudopodia enabled. |
| *Spumella rivalis* Boenigk et Findenig |   (Fig. 3b. *S. rivalis* strian AR4A6. in Findenig et al. 2010)    (Fig. 1D. *S. rivalis*. in Grossmann et al. 2016) | 2.7-3.9 in diameter | | | 2 times longer than cell body | 0.7-1 of the cell body. | In Findenig et al. (2010)  - Stomatocyst is spherical (3.91-6.1 µm in diameter) with low rounded marginal rim collar  In Grossmann et al. (2016)  - Cells are spherical, elongated or posteriorly pointed shape.  - Two unequal flagella. |
| *Spumella sphaerophora* (Skuja) Mignot | *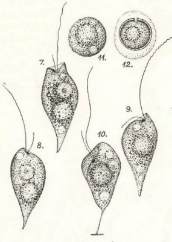*  (Fig. 7-12. *H. aphaerophora* n. sp. 7-9; free-swimming, 10; fixed, 11 and 12; formation of the cyst.)  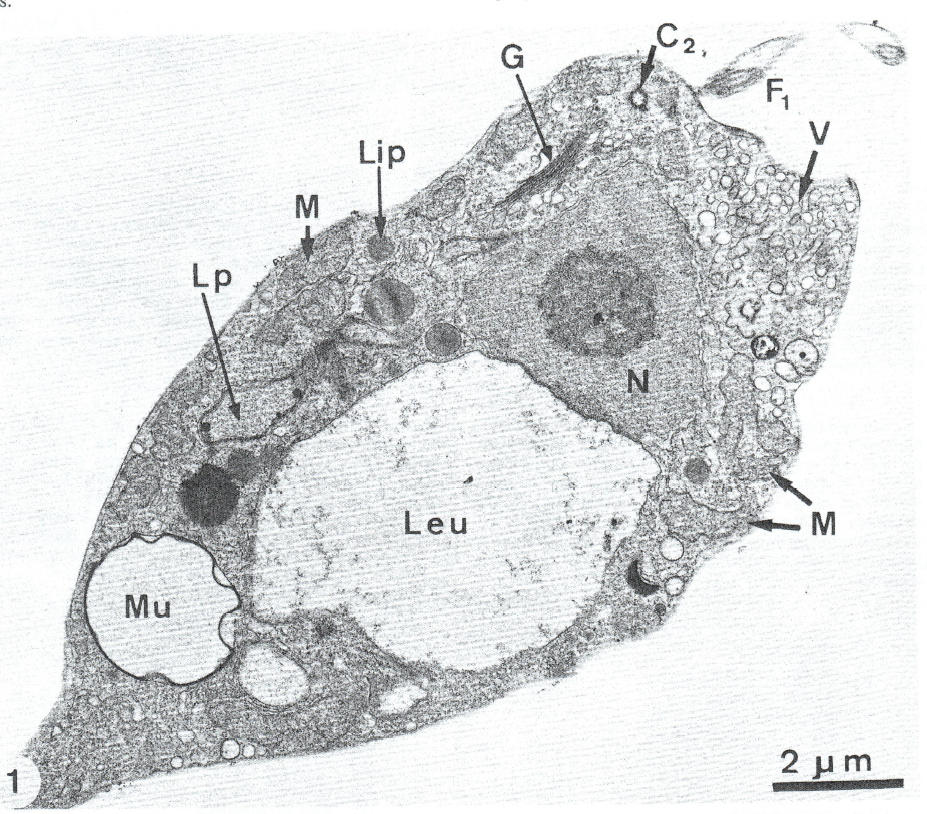  (Fig. 1. Longitudinal section showing the general organization of the cell. Note the eccentric arrangement of the Flagella (F1, C2), the abundance of vesicles (V) near the vestibule, the voluminous leucosine vacuole (Leu) in the median region and behind a curious mucocysts (MU). in Mignot 1977)  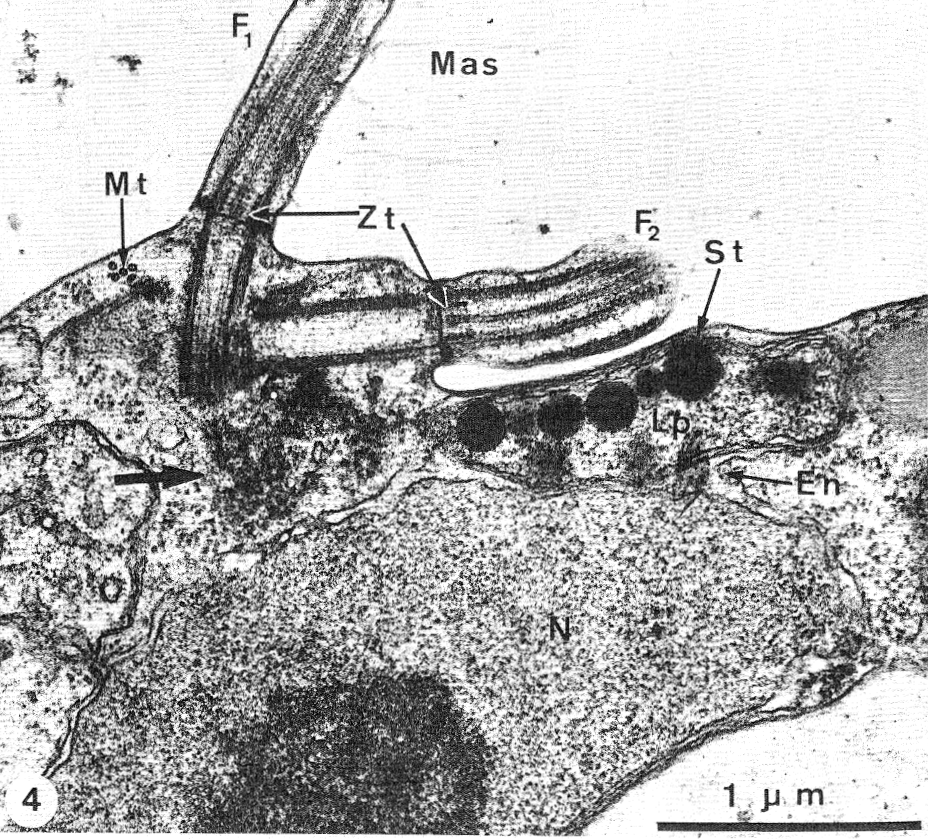  (Fig. 4. Longitudinal section of the apical cell part and passing through the base of the 2 flagella. On the edge of the main flagellum (F1) we see some mastigoneme (Mas). Under the secondary flagellum (F2) along the bottom of the vestibular cup, we see a lobe of the leucoplast (Lp) surrounded by the nuclear envelope (En): and containing the stigma (St). Between the base of the kinetosome and the nucleus appears an important protein condensation (arrow). The microtubules under dandruff (Mt) detach towards the rear of the kinetosome. In both flagella, an internal spiral sleeve placed just above the septum marks the transition zone (Zt)) |  | |  |  |  | In Skuja (1956)  - Cells are ovate, pyriform or the anterior part of the cell obliquely truncate (10-25 µm in length, 5-13 µm in width)  - Attached to substratum using plasmatic stalk (half of the cell body in length)  -Feeding bacteria and small algae.  -Having red stigma  - Stomatocyst is spherical (9-11 µm in diameter) and thickening region around the pore.  - Stomatocyst have thick, colorless and smooth membrane.  In Mignot (1977)  - Cells are pyriform (15-20 µm in length, 6-8 µm in width)  - Having stigma |
| *Spumella termo* (Müller) Hänel | 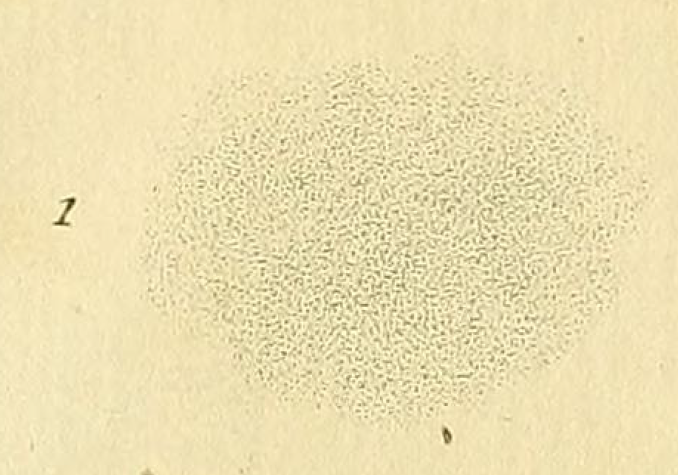  (Fig. 1. *M. termo.* Plate 1 in Müller 1786)  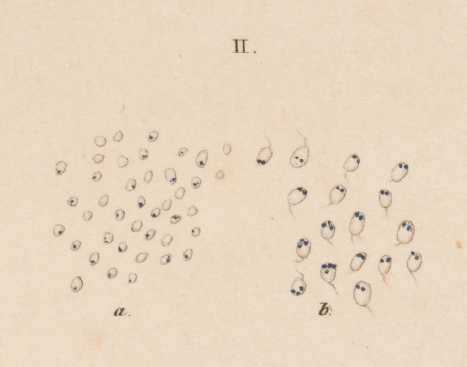  (Fig. 2. *Monas termo.* Plate 1 in Ehrenberg 1838)  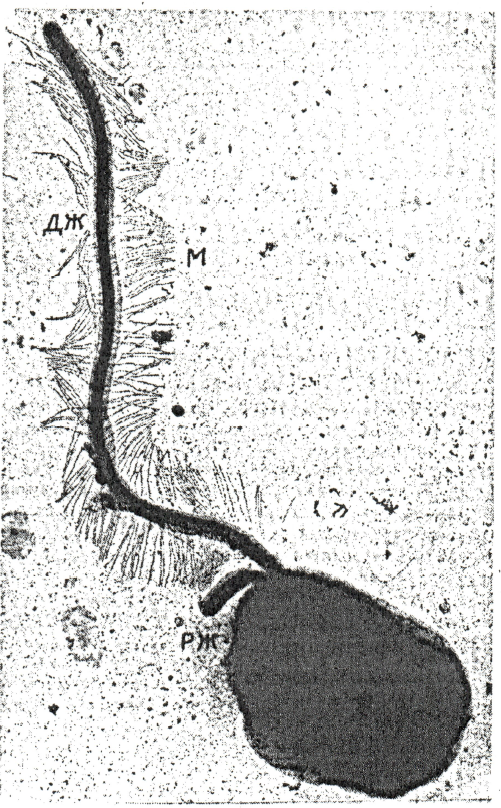  (Fig. 1. *Spumella termo*: vegetative cell in Tanichev 1993)  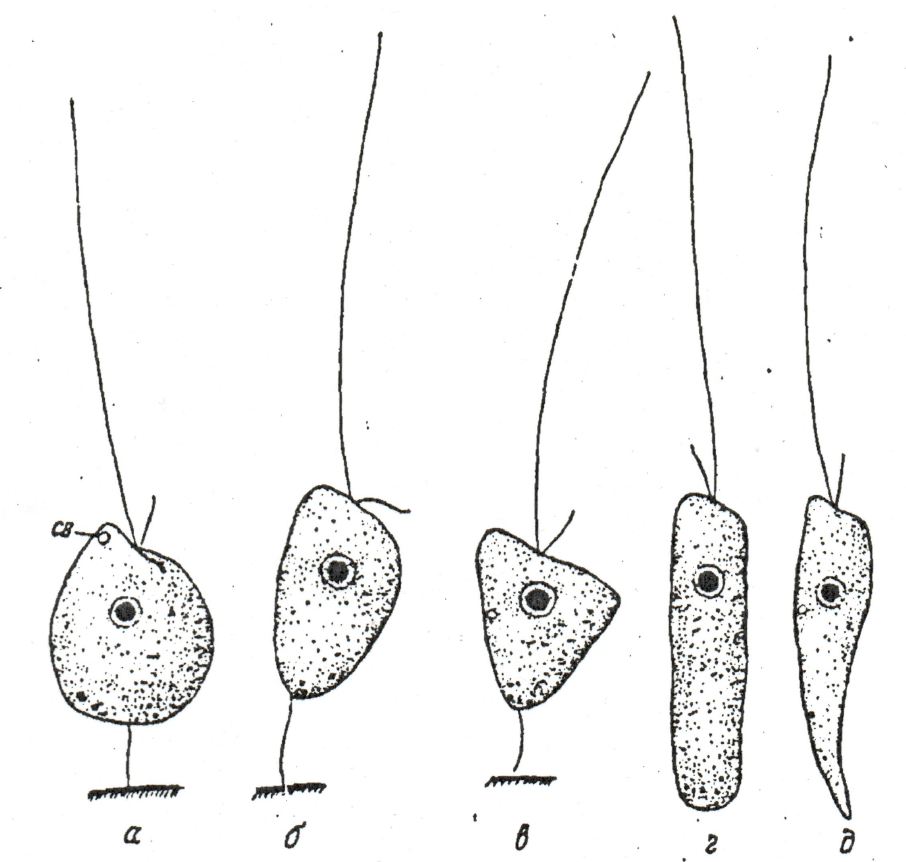  (Fig. 2. Variations in the shape of the cell body in *Spumella termo*: a-в-attached cells. г-д-free-floating cells. in Tanichev 1993)  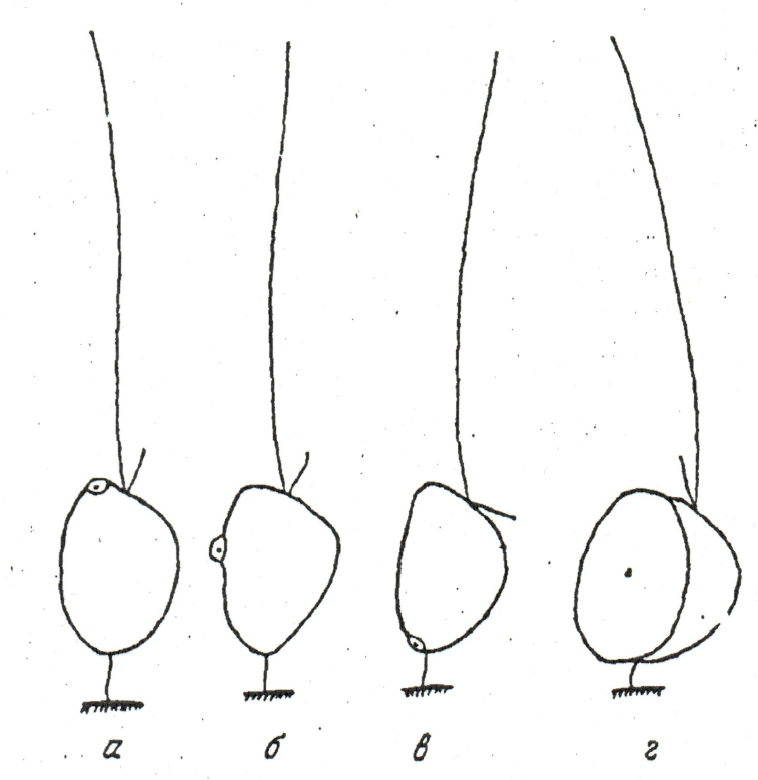  (Fig. 3. Digestive exovacuoles in *Spumella termo*: a-в-moving exovacuole along the body cell. г-macroexovacuole. in Tanichev 1993) |  | |  |  |  | In Müller (1773; 1786):  - Cells are spherical and have gelatin.  In Ehrenberg (1838):  - Cells are spherical, colorless (usually 4.0 to 6.7 µm)  - Consume plant matter as a prey (herbivores).  - Cell have flagellum.  In Tanichev (1977):  - Cells are round, oval or triangular shape and constantly change in shape of the cell body (4-12 µm in length, 6-7 µm in width).  - The all shape of Figure 2 were shown while 20-30 seconds.  - Two unequal flagella (Long: over 2 times longer than cell body, Short: Under 1.5 µm). |
| *Spumella vivipara* (Ehrenberg) Kent | 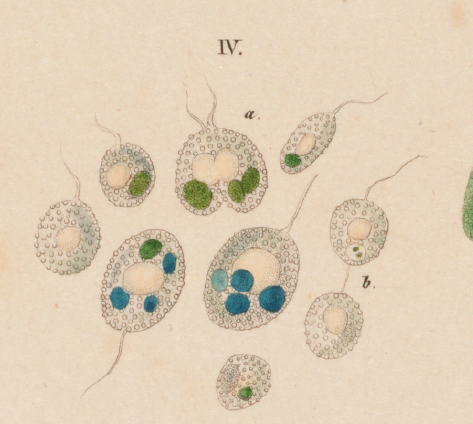  (Fig. 4. *M. vivipara.* The whole group consists of 6 simple and 2 double animals, 450 times diametrically enlarged, 2 of which have absorbed indigo, but 3 contain swallowed individuals of *Chlamydomonas pulvisculus*. One pulled in its trunk. Fig. A. is in the longitudinal division from back to front and has already developed 2 probes in front. Inside one recognizes the division of the middle seminal gland that has begun, and three swallowed dust monads, of which 2 of one half and 1 of the other remain through the division. Fig. B. is almost complete in queer healing. Each half already has its middle gland, but the back half does not yet have a proboscis. The thread-like connecting part is an extended part of the body which, as soon as it tears, contracts, retracts completely, and disappears. Plate 1 in Ehrenberg 1838)  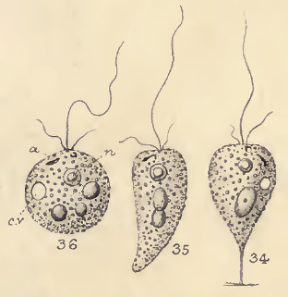  (Fig. 34-36. *Spumella vivipara*. 34 attached, 35 and 36 free-swimming conditions ; at a, eye-like pigment-band or supposed oral aperture, Plate XIV in Kent 1881)  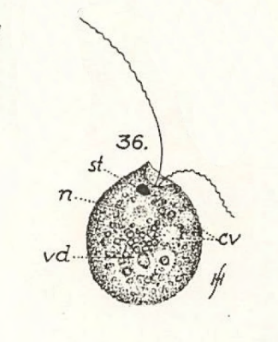  (Fig. 36. *H. vivipara* (Ehrnb.) Pascher. vc; pulsating vacuoles, vd; digestive vacuoles, st; stigma., n; nucleus. Plate LVI in Skuja 1956) |  | |  |  |  | In Ehrenberg (1838):  - Cells are spherical or ovate (10.4-19.2 µm).  - The numerous round granules on the cell surface.  - Feeds on *Chlamydomonas pulvisculus.*  - Cells have flagellum.  In Kent (1880/1881)  - Cells are usually obovate, widest and rounded anteriorly, changeable in shape to ovate, spheroidal, elongate (25.4-41 µm in length)  - Three flagella (One long, two short)  In Skuja 1956  - Cells are rounded or rouded-ovoid (16-25 µm in length, 14-20 µm in width).  - Two unequal flagella (Long: 0.5-1 times longer than cell body, Short: half or little longer than cell body).  - Having relatively large and rounded red stigma.  - Having clear line of the mouth. |
| *Spumella vulgaris* Cienkowsky | 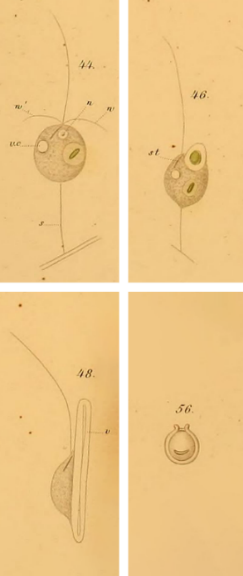 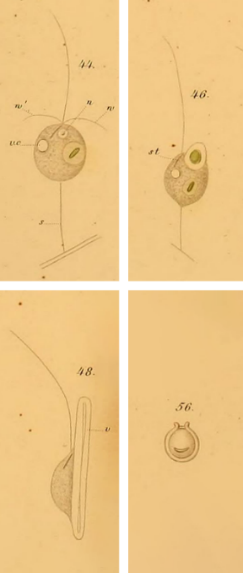  (Fig. 44, 46, 48. *Spumell vulagaris*. 56. Cyst of *Spumella vulgaris*. Plate XXIV in Cienkowsky 1870)  *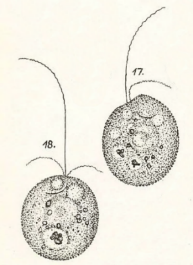*  (Fig. 17-18. *H. vulgaris* (Cienk.) Pascher. Plate LVI in Skuja 1956)    (Figure 4e-f. *Spumella vulgaris* strain 199hm (SAG2322) in Findenig et al. 2010)    (Figure 5b. *Spumella vulgaris* in Findenig et al. 2010) |  | |  |  |  | In Cienkowsky(1870):  - Cells are spherical or oval (14-16 µm).  - Two unequal flagella.  - Consume algal spore, fungal conidia, starch grain.  - The cyst (12 µm) is globule with low collar.  - Having stick in anterior part.  In Skuja (1956)  - Cells are more or less spherical in floating state or rounded egg shaped, obovate in fixed state (15-25 µm in length, 14-22 µm in width).  - Two unequal flagella (Long: 1-2 times longer than cell length, Short: 0.33-0.5 of the cell length).  - Having clear line of the mouth.  In Findenig et al. (2010):  - Cells are usually spherical, elongated or posteriorly pointed (3.2-5.9 µm in diameter)  - Two flagella (Long: 2 times longer than cell body, Short: 0.7-1 times longer than cell body)  - Stomatocysts is spherical (6.1-9.5 µm in diameter)  - Surface of stomatocyst is smooth  - Slightly conical collar (2.2-3.1 µm in diameter, 0.3-0.4 µm in height)  - Planar annulus around the pore of stomatocyst |
